# COP1, a negative regulator of photomorphogenesis, positively regulates plant disease resistance via double-stranded RNA binding proteins

**DOI:** 10.1371/journal.ppat.1006894

**Published:** 2018-03-07

**Authors:** Gah-Hyun Lim, Timothy Hoey, Shifeng Zhu, Marion Clavel, Keshun Yu, Duroy Navarre, Aardra Kachroo, Jean-Marc Deragon, Pradeep Kachroo

**Affiliations:** 1 Department of Plant Pathology, University of Kentucky, Lexington, KY, United States of America; 2 Université de Perpignan Via Domitia, CNRS UMR5096 LGDP, Perpignan, France; 3 U.S. Department of Agriculture–Agricultural Research Service, Washington State University, Prosser, WA, United States of America; Institute of Microbiology, CHINA

## Abstract

The E3 ubiquitin ligase COP1 (Constitutive Photomorphogenesis 1) is a well known component of the light-mediated plant development that acts as a repressor of photomorphogenesis. Here we show that COP1 positively regulates defense against turnip crinkle virus (TCV) and *avrRPM1* bacteria by contributing to stability of resistance (R) protein HRT and RPM1, respectively. HRT and RPM1 levels and thereby pathogen resistance is significantly reduced in the *cop1* mutant background. Notably, the levels of at least two double-stranded RNA binding (DRB) proteins DRB1 and DRB4 are reduced in the *cop1* mutant background suggesting that COP1 affects HRT stability via its effect on the DRB proteins. Indeed, a mutation in either *drb1* or *drb4* resulted in degradation of HRT. In contrast to COP1, a multi-subunit E3 ligase encoded by anaphase-promoting complex (APC) 10 negatively regulates DRB4 and TCV resistance but had no effect on DRB1 levels. We propose that COP1-mediated positive regulation of HRT is dependent on a balance between COP1 and negative regulators that target DRB1 and DRB4.

## Author summary

Plants must constantly regulate the allocation of resources between photomorphogenesis and defense signaling. Although light is known to influence plant defense, the underlying mechanisms remain largely unknown. Here we show that light plays specific and direct signaling roles in plant defense. Specifically, a positive role for COP1 E3 ligase, an important regulator of photomorphogenesis, in plant defense is demonstrated. We further show that COP1 regulates the levels of double-stranded RNA binding proteins DRB1 and DRB4, which in turn regulate the levels of resistance protein HRT that confers resistance against turnip crinkle virus. In contrast to COP1, a multi-subunit E3 ligase encoded by anaphase-promoting complex (APC) 10 negatively regulates DRB4 but had no effect on DRB1 levels. Together, these results suggest that COP1-mediated positive regulation of HRT is dependent on a balance between COP1 and negative regulators that target DRB1 and DRB4.

## Introduction

Resistance (*R*) protein-mediated immunity is induced when a strain-specific avirulence (avr) protein from the pathogen associates with a cognate plant R protein [1]. Induction of R-mediated responses is often accompanied by the formation of a hypersensitive response (HR), a form of programmed cell death resulting in necrotic lesions at the site of pathogen entry [2]. HR is one of the first visible manifestations of pathogen-induced host defenses and is thought to help prevent pathogen multiplication and spread. Plants lacking cognate R proteins can activate the less robust basal defense response, also known as pathogen-associated molecular patterns (PAMPs)-triggered immunity (PTI). In case of bacterial and fungal pathogens, PTI involves recognition of PAMPS by the host encoded pattern recognition receptors. The basal defense response against viral pathogens involves activation of the host RNA silencing pathway, which prevents viral replication and targets viral RNA for degradation (reviewed in [3–5].

Viruses have evolved to express suppressors that target host RNA silencing components and thereby ensure replication in the host [3–5]. Interestingly, in many cases these suppressors of RNA silencing also act as avr factors and their interaction with the host R proteins leads to activation of defense responses. For example, the Arabidopsis R protein HRT [Hypersensitive response (HR) to *Turnip crinkle virus* (TCV) is activated by TCV coat protein (CP) [6, 7], which is a potent suppressor of the host RNA silencing pathway [8, 9]. However, the activation of HRT does not require silencing suppressor function since CP mutants with impaired RNA silencing suppressor activity can elicit normal HR to TCV [10]. Conversely, CP mutant R8A is a functional RNA silencing suppressor that is unable to induce normal HR on Di-17 plants and therefore is virulent on Di-17. Although RNA silencing suppressor and avr activities of CP function independent of each other, the host RNA silencing components are intricately involved in HRT-mediated resistance signaling. This includes double stranded RNA binding protein (DRB) 4, which is required for the post-translational stability of HRT and thereby HRT-mediated HR and resistance to TCV. The loss-of-function mutant *drb4* supports increased replication of TCV on the inoculated leaves of *HRT* containing plants and systemic spread to uninoculated parts [10]. Notably, *HRT drb4* or other *HRT* containing susceptible genotypes (like *HRT sid2*, *HRT eds1*) do not accumulate viral specific small RNAs regardless of TCV levels in their inoculated leaves [10]. This suggests that R-mediated signaling recruits components of the RNA silencing pathway but does not activate the RNA silencing pathway to target viral RNA.

HRT is a coiled coil (CC)- nucleotide binding site (NBS)- leucine rich repeat (LRR) type R protein that is activated in the presence of CP [6, 7, 11, 12], although a direct interaction between HRT and TCV CP has not been demonstrated [13]. While HRT is sufficient for HR formation, resistance to TCV is dependent on HRT and a recessive allele at a second locus, designated *rrt* (regulates resistance to TCV) [14]. Resistance to TCV is also dependent on the SA pathway [11, 14, 15]. Among various components of the SA pathway that regulate HRT-mediated resistance to TCV, enhanced disease susceptibility (EDS) 1, which interacts with HRT, is required for potentiation of CP-triggered HR [13]. HRT is one of the few CC-NBS-LRR proteins that has a direct dependence on EDS1. HRT also interacts with CRT1 (Compromised for Recognition of TCV; [16, 17]) but unlike EDS1, CRT1 is not associated with activation of HR [10]. Interestingly, HRT-DRB4 complex, but not HRT-EDS1 or HRT-CRT1 dissociates in the presence of CP [10], and might play a role in activation of HRT. Besides DRB4, the Arabidopsis genome encodes four other DRB proteins which have been characterized for their roles in RNA biology. Among these DRB1 and DRB4 facilitate DCL1- and DCL4-mediated synthesis of miRNA and *trans-*acting siRNAs (tasiRNAs), respectively [18, 19]. DRB2 is also involved in the biogenesis of specific miRNA subsets [20] and DRB3 and DRB5 are thought to function in the same non-canonical miRNA pathway as DRB2 [20].

HRT-mediated resistance signaling is also dependent on blue-light photoreceptors [15, 21] and of these knocking out CRY2 and PHOT2 results in degradation of HRT. Likewise, blue-light mediated degradation of CRY2 is also associated with the degradation of HRT [15, 21]. HRT does not interact with CRY2 but it does interact with CRY2- and PHOT2-interacting protein COP1 (Constitutive Photomorphogenic 1) [15]. COP1 is an E3 ubiquitin ligase which negatively regulates photomorphogenesis [22]. Furthermore, HRT was degraded in a 26S proteasome-specific manner and pretreatment with MG132 inhibited degradation of HRT [15]. Together, these results suggested that COP1 could be responsible for degradation of HRT.

Here, we examined the role of COP1 in HRT-mediated resistance signaling. Surprisingly, we find that COP1 positively regulates HRT levels, and those of DRB1 and DRB4. Consistent with these results both DRB1 and DRB4 are required for resistance against TCV. In contrast to COP1, a multi-subunit E3 ligase encoded by anaphase-promoting complex (APC) 10 negatively regulates DRB4 and TCV resistance but had no effect on DRB1 levels. Our results suggest that COP1-mediated positive regulation of HRT is dependent on a balance between COP1 and negative regulators that target DRB1 and DRB4.

## Results

### COP1 is required for HRT-mediated resistance to TCV

**Fig 1:**
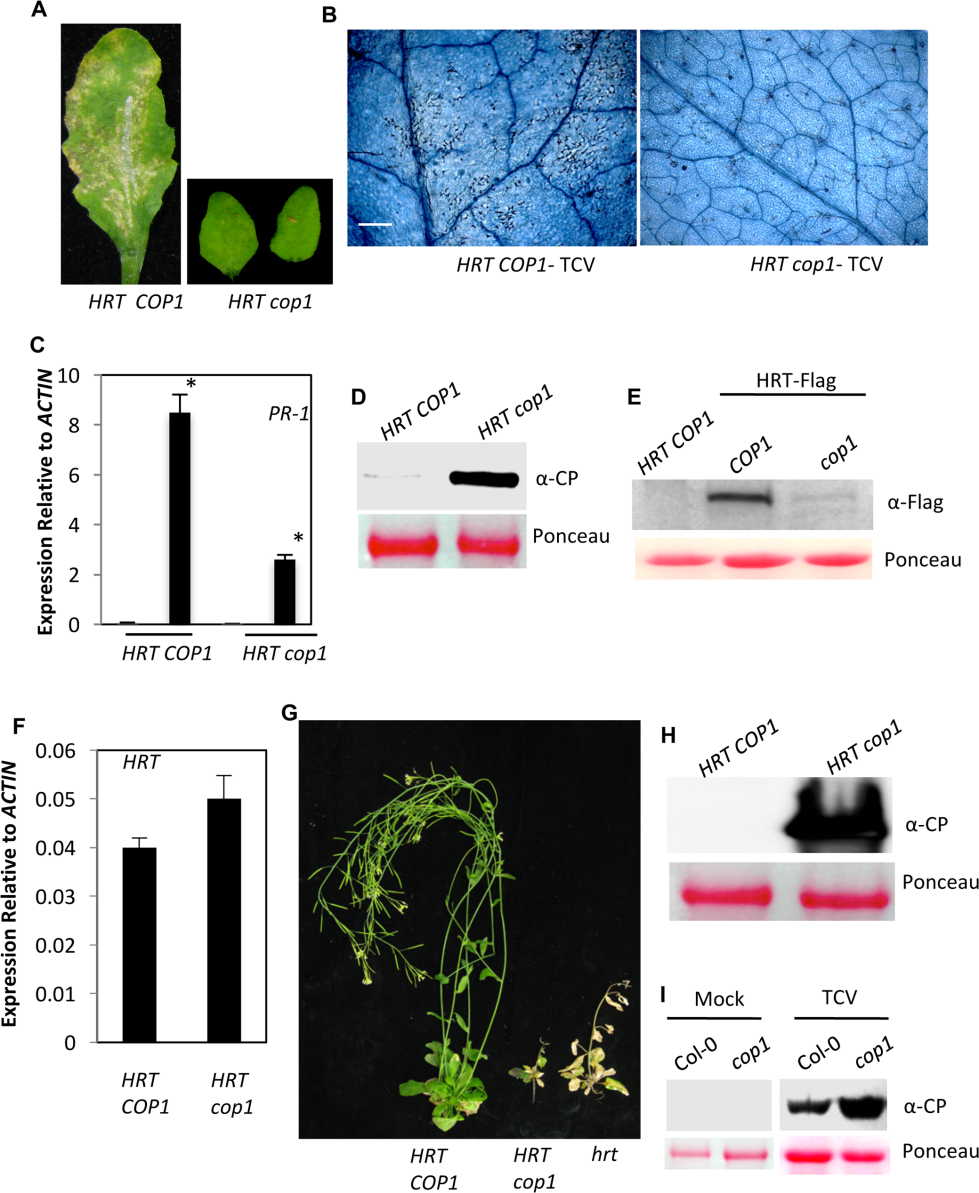
COP1 is a positive regulator of HRT-mediated defense against TCV. (**A**) HR formation in TCV-inoculated *HRT COP1* and *HRT cop1* genotypes at 3 dpi. The HR phenotype was evaluated in ~30 plants that were analyzed in three separate experiments. (**B**) Trypan blue stained leaves showing microscopic cell death phenotype at 3 dpi with TCV. Scale bars, 270 microns. At least six independent leaves were analyzed with similar results. (**C**) Real-time quantitative RT-PCR analysis showing relative expression levels of *PR-1* in mock- and pathogen-inoculated plants. Leaves were sampled 24 h post treatments. The error bars indicate SD (n = 3). Asterisk denotes significant differences from mock-treated leaves (*t* test, P<0.003). Results are representative of two independent experiments. (**D**) Western blot showing relative CP levels in indicated genotypes inoculated with TCV. Leaves were sampled at 3 dpi. Ponceau-S staining of Rubisco was used as the loading control. This experiment was repeated two times with similar results. (**E**) Western blots showing relative levels of HRT-Flag in indicated genotypes expressing *HRT-Flag* transgene. Ponceau-S staining of the Western blots was used as the loading control. This experiment was repeated three times with similar results. (**F**) Real-time quantitative RT-PCR analysis showing relative expression levels of *HRT* in indicated genotypes. The error bars indicate SD (n = 3). Results are representative of two independent experiments. (**G**) Typical morphological phenotypes of TCV inoculated *HRT COP1* (Di-17 ecotype), *HRT cop1* and *hrt* (Col-0 ecotype) plants. Plants were photographed at 18 dpi. (**H**) Western blot showing relative CP levels in distal bolt tissues of indicated genotypes. Plants were inoculated with TCV and the distal uninoculated tissues were sampled at 3 dpi. Ponceau-S staining of the western blot was used as the loading control. This experiment was repeated two times with similar results. (**I**) Western blot showing relative CP levels in mock- and TCV-inoculated genotypes. Plants were inoculated with buffer or TCV and the inoculated tissues were sampled at 3 dpi. Ponceau-S staining of the western blot was used as the loading control. This experiment was repeated two times with similar results.

Interaction between HRT and COP1, together with 26S proteasome-mediated degradation of HRT, suggested that COP1 might be responsible for degradation of HRT. To test this hypothesis, we crossed *cop1-6* plants (Col-0 background, susceptible to TCV, contains recessive allele of the *R* gene *hrt*) with Di-17 (TCV resistant ecotype, contains the *R* gene *HRT*). Similar to Di-17 plants, the F_2_ progeny from the Di-17 x *cop1* cross containing at least one copy of *HRT* and wild-type allele of *COP1* developed visible and microscopic HR following TCV infection (Fig 1A and 1B). Interestingly, in contrast, all *HRT/- cop1/cop1* F2 progeny showed absence of visible or microscopic HR lesions (Fig 1A and 1B), which correlated with their reduced *PR-1* expression (Fig 1C). Furthermore, compared to *HRT COP1* plants, the *HRT cop1* plants supported increased replication of TCV (Fig 1D). Analysis of HRT levels revealed significantly reduced HRT protein in *HRT-Flag cop1* plants as compared to *HRT-Flag COP1* plants (Fig 1E), even though *HRT* transcript levels in *HRT cop1* plants were comparable or higher compared to those in *HRT COP1* plants (Fig 1F). This suggested that lack of COP1 affected HRT protein stability. Next, we evaluated the segregation of resistant plants in Di-17 x *cop1* F2 population. All *hrt/hrt* and ~75% of *HRT*/- (homo/heterozygous for *HRT*) of F_2_ progeny from a Di-17 x Col-0 cross showed typical crinkled leaves and drooping bolt phenotypes associated with susceptible plants. Only 25% (homo/heterozygous for *HRT*, but homozygous for *rrt*) of these HR-developing progeny were able to resist TCV infection and did not allow the virus to spread into uninoculated tissues. In contrast, all *HRT cop1* progeny showed susceptible phenotype suggesting that COP1 positively regulated HRT-mediated resistance to TCV (Fig 1G and 1H and S2 Table). Likewise, COP1 was also required for basal resistance to TCV; in comparison to Col-0, the *cop1* plants accumulated more TCV CP in their inoculated leaves (Fig 1I).

### COP1 positively regulates RPM1 levels

**Fig 2:**
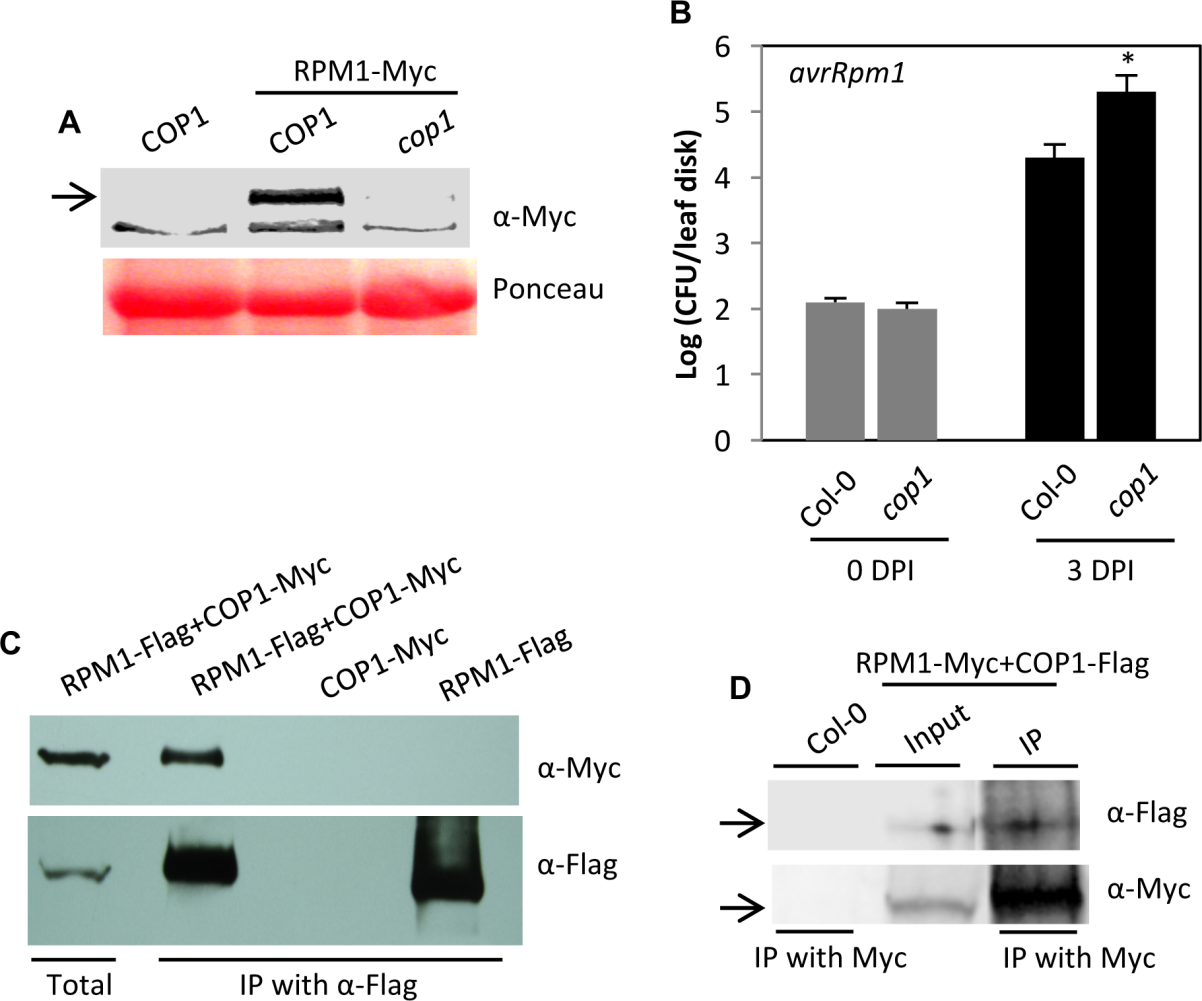
COP1 is a positive regulator of RPM1-mediated defense against *Pst*. (**A**) Western blot showing relative levels of RPM1-Myc in wild-type and *cop1* plants. Ponceau-S staining of the western blots was used as the loading control. Arrow indicates the target protein corresponding to the indicated antibody. This experiment was repeated three times with similar results. (**B**) Growth of *Pst avrRpm1* on *cop1*. Error bars indicate SD. Asterisks indicate data statistically significant from that of control (Col-0) (P<0.05, n = 4). (**C**) IP of COP1-Myc with RPM1-Flag. *N*. *benthamiana* plants were agroinfiltrated and immunoprecipitated proteins were analyzed with α-Myc and α-Flag. This experiment was repeated twice with similar results. (**D**) IP of COP1-Flag with RPM1-Myc. *RPM1-Myc* and *COP1-Flag* were expressed under their native or 35S promoters, respectively. Arrows indicate the target protein corresponding to the indicated antibody. The immunoprecipitated proteins were analyzed with α-Myc and α-Flag and this experiment was repeated twice with similar results.

To determine if COP1 regulates levels of other R proteins, we analyzed the role of COP1 in RPM1-mediated resistance. The R protein RPM1 confers resistance to the avrRpm1 expressing strain of *Pseudomonas syringae* pv. *tomato* (*Pst*) [23]. To this end, we crossed *cop1* with Col-0 plants expressing RPM1-Myc under its native promoter and generated *cop1 RPM1-Myc* plants. Interestingly, RPM-Myc protein levels were significantly reduced in the *cop1* mutant background (Fig 2A), even though *RPM1* transcript levels in the *cop1* background were comparable to those in *COP1* plants (S1A Fig). This suggested that lack of COP1 affected RPM1 protein stability. Consistent with this phenotype, the *cop1* plants showed increased susceptibility to *avrRpm1 Pst* (Fig 2B). Next, we assayed the interaction between COP1 and RPM1 using bi-molecular fluorescence complementation (BiFC) assay in *Nicotiana benthamiana* and co-immunoprecipitation (IP) assays in *N*. *benthamiana* and Arabidopsis. RPM1 did interact with COP1 and this interaction was primarily observed in the cytoplasm (S1B Fig). The BiFC result was verified using IP assays of transiently expressed proteins in *N*. *benthamiana* (Fig 2C) and confirmed in the native Arabidopsis system (Fig 2D). Together, these results suggested that COP1 positively regulates RPM1 levels.

### COP1 regulates stability of DRB1 and DRB4

**Fig 3:**
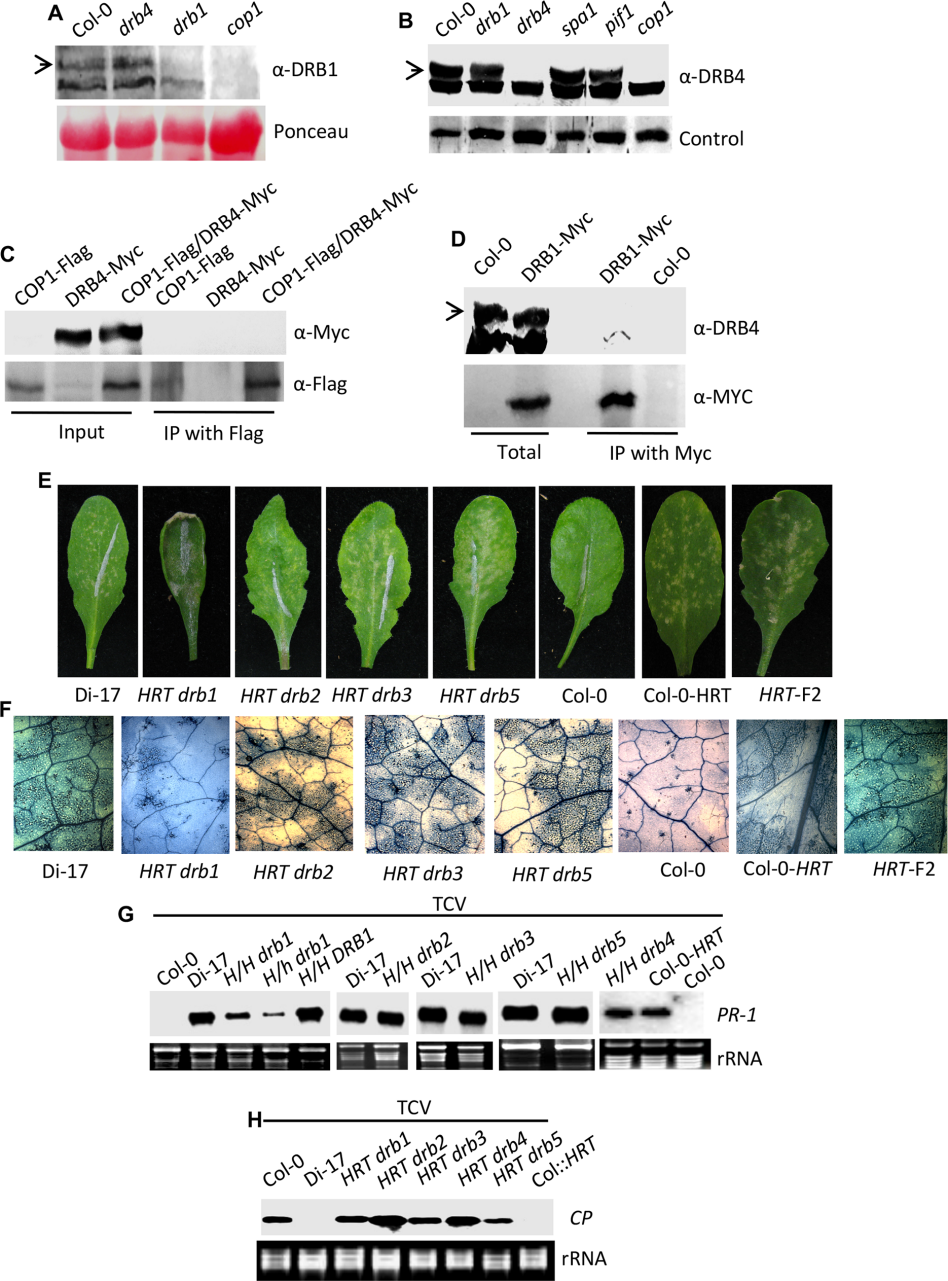
COP1 regulates DRB1 and DRB4 levels and thereby TCV resistance. (**A-B**) Western blots showing relative levels of DRB1 (**A**), and DRB4 (**B**) in indicated genotypes. Ponceau-S staining of the Western blots was used as the loading control. Arrows indicate the target protein corresponding to the indicated antibody. This experiment was repeated three times with similar results. (**C**) Co-immunoprecipitation (IP) assay carried out between DRB4-Myc and COP1-Flag proteins. *DRB4* and *COP1* were expressed under their native or 35S promoters, respectively, and the transgenic plants were crossed to create a line co-expressing both the proteins. The immunoprecipitated proteins were analyzed with α-Myc and α-Flag and this experiment was repeated twice with similar results. (**D**) IP assay carried out between DRB1-Myc and DRB4 proteins. DRB1-Myc was expressed under its native promoter and the immunoprecipitated proteins were analyzed with α-Myc and α-DRB4 and this experiment was repeated twice with similar results. (**E**) HR formation in TCV-inoculated Di-17, Col-0, Col-0 containing an introgressed copy of HRT and *HRT drb* genotypes at 3 dpi. The HR phenotype of *HRT drb* plants was evaluated in ~40–50 plants per genotype that were analyzed in five to seven separate experiments. (**F**) Trypan blue stained leaves showing microscopic cell death phenotype at 3 dpi with TCV. Scale bars, 270 microns. At least five independent leaves were analyzed with similar results. (**G**) RNA gel blot analysis showing expression of *PR-1* in indicated genotypes after inoculation with TCV. Total RNA was extracted from inoculated leaves at 3 dpi. Ethidium bromide staining of rRNA was used as the loading control. *H/H* and *H/h* indicate plants homozygous or heterozygous for *HRT*, respectively. The experiment was repeated twice with similar results. (**H**) RNA gel blot analysis showing relative levels of genomic *CP* RNA in indicated genotypes inoculated with TCV. Leaves were sampled at 3 dpi. Ethidium bromide staining of rRNA was used as the loading control. This experiment was repeated three times with similar results.

Recent results showing that COP1 and DRB4 positively regulate DRB1 [24] and HRT levels [10], respectively, prompted us to analyze the relationship between COP1, DRB1 and DRB4. Interestingly, *cop1* plants contained reduced levels of both DRB1 and DRB4 (Fig 3A and 3B). However, the *cop1* plants accumulated normal levels of DRB2 (S1C Fig). The evaluation of DRB3 and DRB5 levels was limited by the inability of DRB3 and DRB5 specific antibodies to detect distinct bands in protein gel-blot analyses. A loss-of-function mutations in two proteins (SPA1 or PIF1) that interact with COP1 and contribute to COP1 activity, did not affect DRB4 levels (Fig 3B). This suggests that COP1 protein that was not in the COP1-SPA1-PIF1 complex contributed to DRB4 protein levels. To determine if this regulation of DRB4 levels involved interactions between COP1 and DRB4, we generated Arabidopsis plants coexpressing COP1-Flag and DRB4-Myc and used these for IP assays. No interaction was detected between COP1 and DRB4 (Fig 3C). Thus, unlike DRB1 [24], COP1-mediated regulation of DRB4 is unlikely to be the result of direct/indirect physical associations between these proteins. Furthermore, the *drb1* and *drb4* mutants contained wild-type-like levels of the reciprocal proteins (Fig 3A and 3B) and DRB1 did not associate with DRB4 (Fig 3D). Together, these results suggested that COP1-mediated regulation of DRB1 and DRB4 levels likely involves independent processes.

Earlier we showed that DRB4 is required for HRT-mediated resistance to TCV signaling [10]. To test if DRB1, and other DRB proteins, are also required for HR and/or resistance to TCV, we generated homozygous mutant lines in all DRB proteins. All the knock-out (KO) lines used here were characterized in a previous study [25] (S1 Table). As shown before, *drb1* plants showed short-stature and *drb4* plants showed the zippy (narrow leaves) phenotype (S2A Fig). Next, we crossed *drb* plants (Col-0 background) with Di-17 (TCV resistant ecotype). The F_2_ progeny from a Di-17 x Col-0 control cross or *HRT* introgressed into Col-0 background (backcrossed 8 times) were used as controls and both these genotypes developed visible and microscopic HR following TCV infection (Fig 3E). Likewise, all *HRT/- drb/drb* F2 progeny, except *HRT/- drb1/drb1*, developed normal HR (Fig 3E and 3F) and induced wild-type-like *PR-1* gene expression (Fig 3G). In contrast, *HRT/- drb1* plants showed fewer microscopic HR lesions (Fig 3F), which correlated with their reduced *PR-1* expression (Fig 3G). Notably, all *HRT drb* genotypes supported increased replication of TCV compared to Di-17 or Col-0-*HRT* plants (Fig 3H). Together, these results suggested that while all DRBs were required for HRT-mediated local resistance to TCV, only DRB1 contributed to HR development in response to TCV infection.

Next, we evaluated the segregation of resistant plants in Di-17 x *drb* F2 population. All *hrt/hrt* and ~75% of *HRT*/- (homo/heterozygous for *HRT*) of F_2_ progeny from a Di-17 x Col-0 cross showed typical crinkled leaf and drooping bolt phenotypes associated with TCV susceptibility. Only 25% (homo/heterozygous for *HRT*, but homozygous for *rrt*) of these HR-developing progeny were able to resist TCV infection and did not exhibit virus spread to uninoculated tissues. Evaluation of genetic segregation in Di-17 x *drb2* and Di-17 x *drb3* crosses showed statistically significant deviation from Mendelian segregation; all *HRT drb2* and *HRT drb3* plants showed typical susceptible symptoms suggesting that DRB2 and DRB3 proteins were required for resistance to TCV (S2B Fig, S2 Table). The involvement of *DRB5* in TCV resistance could not be fully ascertained since *DRB5* is located 1 Mb North of *HRT* resulting in skewed segregation in the progeny of Di-17 x *drb5* cross (S2 Table). Nonetheless, all *HRT drb5* progeny showed susceptible phenotype suggesting that DRB5 was also required for HRT-mediated resistance to TCV (S2B Fig, S2 Table). Likewise, involvement of DRB1 in the resistance response could not be firmly established since *HRT drb1* F2 plants were difficult to inoculate due to their curled leaves and often yielded ~5–12% resistant plants (S2 Table). The requirement of *DRB1* in HRT-mediated resistance was further assessed using *DRB1* knock-down plants (see below).

### DRB proteins are required for HRT stability

**Fig 4:**
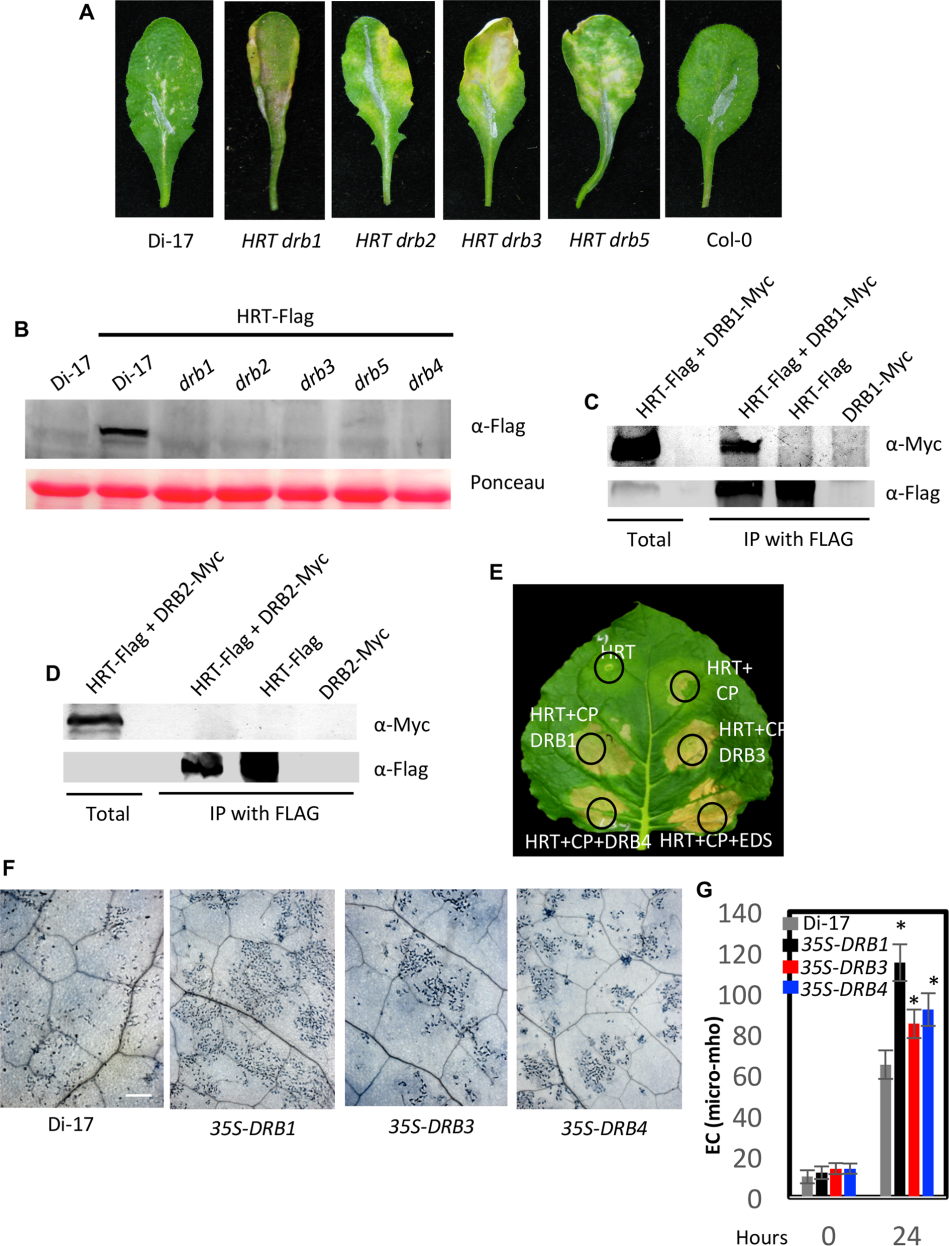
DRB proteins are required for the stability of HRT. (**A**) HR formation in TCV-inoculated Di-17, Col-0 and *HRT drb* genotypes at 10 dpi. The HR phenotype was evaluated in ~20–30 plants that were analyzed in four separate experiments. (**B**) Western blots showing relative levels of HRT-Flag in Di-17 and *drb* genotypes expressing *HRT-Flag* transgene. Ponceau-S staining of the Western blots was used as the loading control. This experiment was repeated three times with similar results. (**C** and **D**) IP of DRB1-Myc (**C**) and DRB2-Myc (**D**) with HRT-Flag. All proteins were expressed under their respective native promoters in Arabidopsis. The immunoprecipitated proteins were analyzed with α-Myc and α-Flag and this experiment was repeated twice with similar results. (**E**) Visual phenotype of *Nicotiana benthamiana* leaves expressing indicated proteins. Agroinfiltration was used to express HRT, CP, EDS1 (E90-At3g48090), and DRB1, DRB3 or DRB4 proteins. The leaf was photographed at 4 days post treatment. (**F**) Trypan blue stained leaves of Di-17 and transgenic plants overexpressing *DRB1*, *DRB3* and *DRB4* in Di-17 background. The plants were inoculated with TCV and the inoculated leaves were sampled at 36 h post inoculation. Scale bars, 270 microns. At least four independent leaves were analyzed with similar results. (**G**) Electrolyte leakage in genotypes shown in F. The leaves were sampled at 0 and 24 h post TCV inoculation. Error bars represent SD. Asterisks indicate data statistically significant from that of control (Col-0) (P<0.05, n = 4).

Earlier we showed that degradation of HRT was associated with a spreading HR phenotype wherein HR lesions coalesced resulting in prominent chlorosis [10]. This was seen in *HRT drb4*, *HRT crt1*, and *HRT cry2* genotypes, all of which showed reduced levels of HRT [10]. Comparison of HR phenotypes in the *HRT drb* genotypes at 10 days post inoculation (dpi) showed pronounced chlorosis on *HRT drb2*, *HRT drb3* and *HRT drb5* but not *HRT drb1* leaves (Fig 4A). Analysis of HRT levels revealed significantly reduced HRT protein in *HRT-Flag drb1*, *HRT-Flag drb2*, *HRT-Flag drb3*, and *HRT-Flag drb5* transgenic plants as compared to *HRT-Flag DRB* plants (Figs 4B and S3A), even though *HRT-Flag* transcript levels in *HRT-Flag drb* plants were comparable to those in wild-type plants (S3B Fig). This suggests that absence of DRB proteins specifically affected HRT protein stability. Together with the spreading HR phenotype of *HRT drb* plants, this suggests that a certain threshold level of HRT is required for proper HR. Clearly, the spreading HR phenotype was HRT-dependent and unrelated to TCV replication because Col-0 plants (*hrt*), which contained the highest levels of TCV in inoculated leaves, did not show spreading lesions/cell death (Figs 3E and 4A). Likewise, *hrt drb* plants did not show HR lesions, and Col-0-*HRT* plants showed Di-17-like non-spreading HR-like lesions (S3C Fig).

To determine if DRB proteins contribute to the stability of HRT via physical interactions with the R protein, we used BiFC assays in *N*. *benthamiana*. HRT did interact with DRB1, DRB3, and DRB5, but not DRB2, and these interactions were primarily observed in the cytoplasm (S3D Fig). The BiFC results were verified using IP assays of transiently expressed proteins in *N*. *benthamiana* (S3E, S3F, S3G and S3H Fig). The IP assays for DRB1 and DRB2 were further confirmed in the native Arabidopsis system where DRB1 and DRB2 proteins were expressed under their native promoters (Fig 4C and 4D and S1 Table). To determine if increased expression of DRB proteins potentiated the activation of HRT, we first monitored the HR phenotype in *N*. *benthamiana* plants transiently co-expressing DRB1, DRB3, or DRB4 with HRT and CP (Fig 4E). As shown earlier, co-expression of HRT and CP triggered nominal cell death, and the presence of EDS1 enhanced this response [13] (Fig 4E). Interestingly, co-expression of DRB1, DRB3 or DRB4 proteins with HRT and CP also enhanced HR (Figs 4E and S3I). To confirm this in the native system we generated Arabidopsis plants overexpressing *DRB1*, *DRB3*, or *DRB4*, in the Di-17 background and evaluated T2 and T3 plants for HR and resistance to TCV. Multiple lines were evaluated for each transgene and at least two lines expressing higher levels of *DRB* transcripts were selected for further analysis (S3J Fig). As observed in transient assays, overexpression of *DRB1*, *DRB3* or *DRB4* resulted in increased cell death response after TCV infection and this phenotype was particularly pronounced in *DRB1* overexpressing plants (Fig 4F and 4G). Notably, this analysis also identified two Di-17 DRB1 lines that showed significantly reduced expression of *DRB1* (#1–1 and 1–8, S3J Fig), likely due to transgene co-suppression. Interestingly, like *HRT drb1*, the *DRB1-1* and *DRB1-8* lines showed impaired HR (S3K Fig), which corresponded to increased susceptibility to TCV (S3L, S3M and S3N Fig). All *35S-DRB* plants showed wild-type-like susceptibility to the virulent TCV strain R8A (S3O–S3Q Fig). Together, these results suggested that DRB proteins are important for stabilizing HRT and that DRB1 plays a more important role in HRT-mediated signaling. This is further consistent with the impaired activation of HRT in *cop1* plants, which contains reduced levels of DRB1 protein.

Since HRT interacts with DRB1, DRB3, DRB4, DRB5 and COP1, it was possible that degradation of HRT in *drb* plants is due to impaired COP1 function. To test this we evaluated photomorphogenesis in *drb* plants. As expected, *cop1* plants were unable to sense light and produced a short hypocotyl when grown in the dark (S4A and S4B Fig). In comparison, wild-type and *drb* mutant plants produced a long hypocotyl in the dark suggesting that *drb* plants are not impaired in the COP1 function (S4A and S4B Fig).

### Viral coat protein prevents HRT-DRB1 complex formation

**Fig 5:**
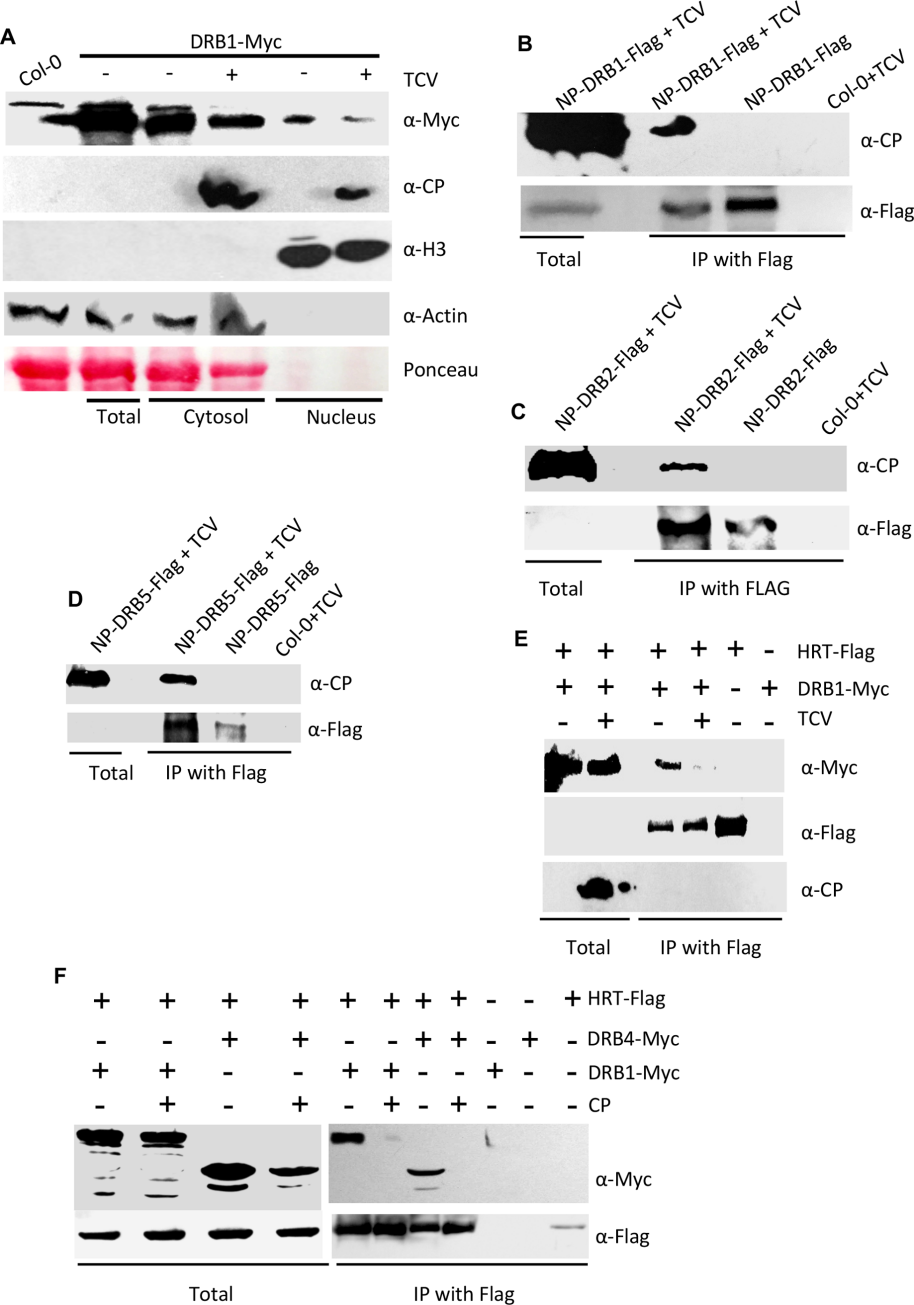
DRB proteins interact with CP. (**A**) Relative levels of DRB1 in nucleus and cytosolic fractions of Arabidopsis plants expressing DRB1-Myc under its self promoter. The blot was sequentially probed with indicated antibodies. Ponceau-S staining of the Western blot was used as the loading control. This experiment was repeated two times with similar results. Fold change, normalized with Rubisco, Actin or H3 proteins, in western blots was quantified using Image Quant software. (**B-D**) Co-IP of DRB1-Flag, (**B**) DRB2-Flag (**C**) and DRB5-Flag (**D**) in the presence of TCV. The transgenic Arabidopsis plants expressing *DRB1-Flag*, *DRB2-Flag*, and *DRB5-Flag* under their respective native promoters (NP) were inoculated with TCV and leaves sampled at 3 dpi were processed for Co-IP. The TCV inoculated Col-0 plants were used as a negative control. These experiments were repeated twice with similar results. (**E and F**) Co-IP of DRB1-Myc or DRB4-Myc with HRT-Flag in the presence or absence of TCV (**E**) or CP (**F**). Arabidopsis expressing DRB1 and HRT under their native promoters were used in **E**. For transient assays shown in **F**, *N*. *benthamiana* plants were agroinfiltrated and immunoprecipitated proteins were analyzed with α-Myc and α-Flag. The experiments shown in **E** and **F** were repeated two times with similar results.

Unlike DRB2, DRB3, or DRB5, the DRB1 protein preferentially localizes to the nucleus in transient assays carried out in *N*. *benthamiana* (S5A Fig). However, DRB1 interacts with HRT in the cytosol (S3D Fig). To follow up on this observation, we assayed the effect of TCV infection on the sub-cellular localization of DRB1. Because we were unable to obtain native promoter-based DRB1-GFP transgenic plants, we assayed localization of DRB1 in transgenic *drb1* plants expressing DRB1-Myc via its native promoter. Surprisingly, unlike our transient localization assays (S5A Fig) and transient assays reported by others [26–28], a significant proportion of DRB1 was detected in the cytosol of Arabidopsis plants (Fig 5A). Notably, the nuclear-cytoplasmic DRB1 levels seen in our study are consistent with an earlier report that evaluated DRB1 levels in Arabidopsis plants [24]. Interestingly, TCV infected plants showed a ~2.34-fold reduction in the nuclear levels of DRB1 (normalized based on H3 levels; Fig 5A), suggesting cytoplasmic relocalization of some nuclear DRB1 in response to TCV infection. Co-expression of CP-RFP with DRB1-GFP in *N*. *benthamiana* also increased the extranuclear localization of DRB1, directing DRB1 and CP to punctate foci in the cytoplasm (shown by arrowheads, S5B Fig). In contrast, CP did not appear to alter the overall nuclear or extra-nuclear levels of DRB2-GFP (S5B Fig). Notably, a small percentage of CP was detected in the nuclear fraction (Fig 5A). This promoted us to assay the interaction between CP and DRB proteins. CP interacted with all DRB proteins in IP assays carried out in Arabidopsis and *N*. *benthamiana* Figs (5B, 5C and 5D and S5C, S5D, S5E and S5F). CP also interacted with DRB4 in the yeast-two hybrid assay (S5G and S5H Fig), suggesting that CP directly associated with DRB4. BiFC assays showed that the interaction between CP and DRB proteins was preferentially observed in inclusion structures that are formed in cells containing CP (S5I Fig). To confirm that nuclear DRB does not associate with CP we assayed the interaction between CP and DRB2 that was directed exclusively to the nucleus (fused with nuclear localization signal, NLS) or the cytosol (fused with nuclear export signal, NES). CP interacted with DRB2-NES (cytosolic DRB2) but not DRB2-NLS (nuclear DRB2) (S5I and S5J Fig), suggesting that the CP-DRB complex occurred only in the cytosol.

Earlier we showed that TCV infection (in Arabidopsis), or CP expression (in *N*. *benthamiana*), increased the cytosolic pool of DRB4 and inhibited the HRT-DRB4 interaction [10]. As shown above CP also increased the cytosolic levels of DRB1 (Figs 5A and S5B). Therefore, we assayed HRT-DRB1 complex formation in the presence or absence of CP. Interestingly, like HRT-DRB4, TCV infection or presence of CP also inhibited the HRT-DRB1 interaction (Fig 5E and 5F). Notably, this was not the case for the HRT-DRB3 interaction (S5K Fig), suggesting that CP-dependent inhibition of HRT-DRB1/DRB4 interactions was not a generalized effect. The CP-dependent dissociation of HRT-DRB1 complex correlated with impaired HR phenotype in *HRT drb1* plants. However, *HRT drb4* showed normal HR at 3 dpi even though CP also inhibited the HRT-DRB4 interaction [10]. This, together with the impaired HR phenotypes of *HRT drb1* and *DRB1* knock-down plants, suggested that DRB1 may be a dominant player in HR formation and thereby activation of HRT. Congruent with this notion, *HRT cop1* plants that lack both DRB1 and DRB4 proteins showed loss of both visible as well as microscopic HR (Fig 1B), suggesting that DRB1 and DRB4 acted additively, with DRB1 played a major role in the activation of HRT.

### Anaphase-promoting complex (APC) 10 negatively regulates DRB4 but does not alter COP1 activity

**Fig 6:**
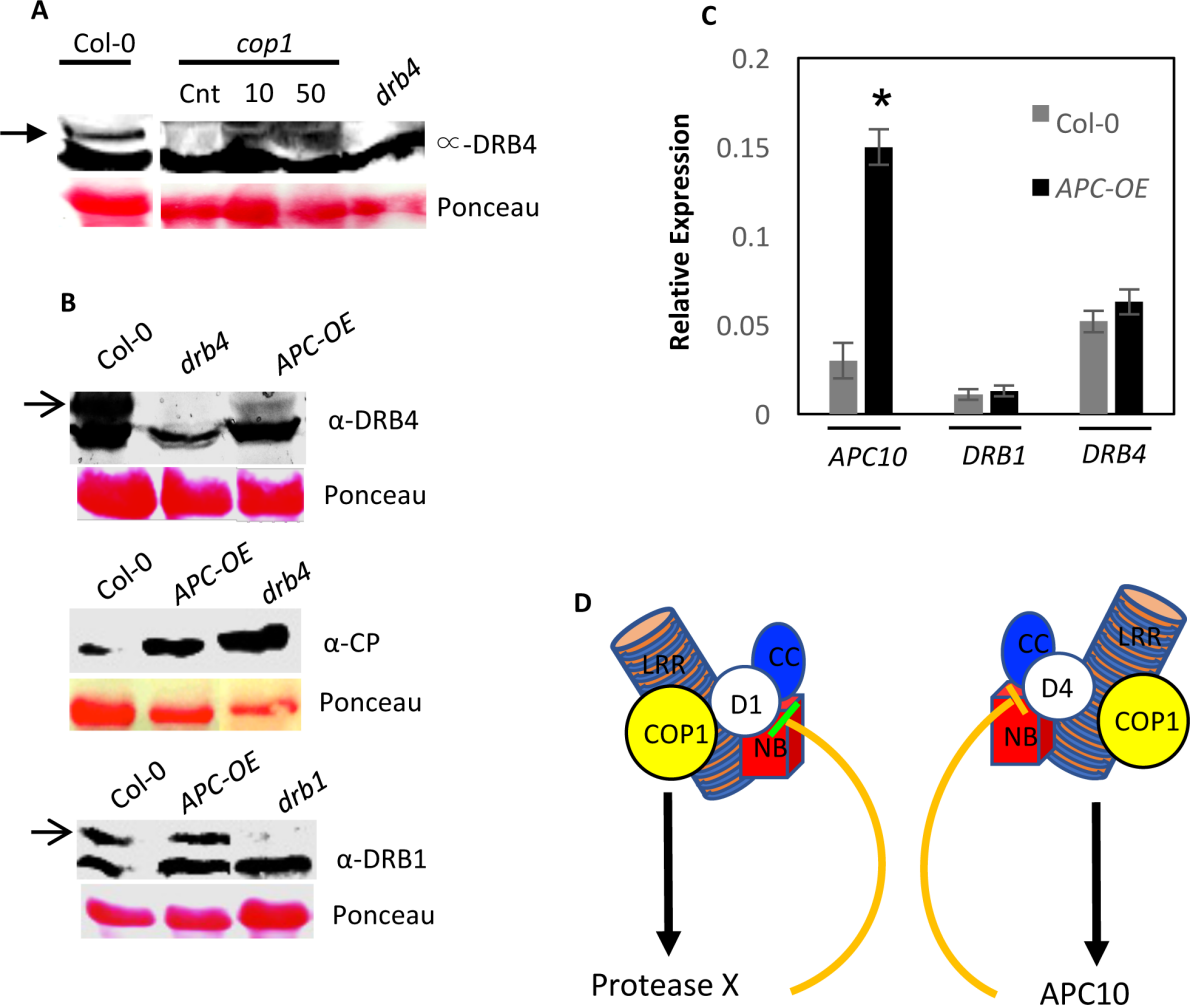
APC10 negatively regulates DRB4 levels. (**A**) Western blot showing DRB4 levels in *cop1* plants infiltrated with 10 or 50 μM MG132. The control (cnt) plants were infiltrated with DMSO and the leaves were sampled 24 h post infiltration. The Col-0 and *drb4* plants were used as additional controls. (**B**) Western blots showing relative levels of DRB4 (upper panel), CP (middle panel) or DRB1 (lower panel) in *APC10* overexpressing (OE) plants. Ponceau-S staining of the Western blots was used as the loading control. This experiment was repeated three times with similar results. (**C**) Quantitative RT-PCR analysis showing relative levels of *APC10*, DRB1 and DRB4 transcripts in wild-type (Col-0) and *APC10* overexpressing (OE) plants. This experiment was repeated twice using two or more independent cDNA preparations as templates. (**D**) Proposed model of regulation of HRT levels by COP1, DRB1 and DRB4 proteins. HRT interact with DRB1 (D1), DRB4 (D4) and COP1 proteins. COP1 interacts with DRB1 [24], but not with DRB4. Moreover, DRB1 and DRB4 do not interact with each other. Although a mutation in either DRB1 or DRB4 results in degradation of HRT, only a mutation in DRB1 abolishes HR to TCV. These observations suggest that DRB1 and DRB4 might form separate complexes with HRT. COP1 was recently shown to stabilize DRB1 by negatively regulating an unknown protease [24]. Likewise, DRB4 was previously shown to interact with APC10 E3 ligase [30], which negatively regulates DRB4 levels. Thus, COP1 might be protecting DRB1 and DRB4 proteins by negatively regulating a putative protease or APC10, respectively. A loss of COP1 will therefore result in the activation of protease or E3 ligase, which in turn will degrade DRB1 and DRB4 proteins, respectively. Alternately, COP1- and APC10-mediated regulation of DRB4 could be independent processes that rely on the relative levels of APC10 in the cell. Together, these results show that components of the RNA silencing pathway and photomorphogenesis are intricately associated with the stability/activation of the R proteins.

To determine if degradation of DRB4 in *cop1* plants occurred in a 26S proteasome-dependent manner, we assayed recovery of DRB4 in the *cop1* plants that were treated with proteasome-specific inhibitor MG132. The *cop1* leaves pretreated with MG132 accumulated significantly higher levels of DRB4 protein (Fig 6A), suggesting that DRB4 in *cop1* plants was degraded in a proteasome-dependent manner. This is further consistent with earlier results showing that APC10 subunit of the anaphase promoting complex (APC) interacted with DRB4 and elevated levels of DRB4 in APC10 RNAi plants suggested that APC10 targeted DRB4 for degradation [29, 30]. To test this further and to investigate relationship between APC10 and COP1, we first analyzed DRB4 levels in *APC10* overexpressing Col-0 plants [31]. As predicted, the *APC10* overexpressing plants showed reduced levels of DRB4, suggesting that the increased expression of *APC10* negatively regulated accumulation of DRB4 (Fig 6B, upper panel). Consistent with this result, the *APC10* overexpressing plants showed increased levels of TCV-CP in their inoculated leaves (Fig 6B, middle panel). Normal *DRB4* transcript in *APC10* overexpressing plants suggested that APC10-mediated negative regulation of DRB4 was a post-translational event (Fig 6C). Overexpression of *APC10* had no effect on DRB1 (Fig 6B, bottom panel). No interaction was detected between APC10 and COP1 (S6A Fig), suggesting that APC10-mediated negative regulation of DRB4 did not involve physical sequestration of COP1. Furthermore, *APC10* overexpressing plants showed wild-type like photomorphogenetic phenotype in light and dark, suggesting that these plants were not altered in the COP1 function (S6B Fig). Analysis of Arabidopsis interactome comprising of predicted or known interactions with COP1, DRB4, and APC10 was unable to identify any proteins that are shared between COP1 and DRB4 or APC10 (S6C Fig). Together, these results suggest that COP1- and APC10-mediated regulation of DRB4 might involve independent processes but the relative levels APC10 play an important role in the stability of DRB4 (Fig 6D), and thereby disease resistance.

## Discussion

The earth’s natural light environment undergoes continuous spatial and temporal fluctuations and living organisms have evolved to regulate their growth and well-being in response to these fluctuations. Plants being sessile have to particularly modify their growth and development for optimized utilization of ambient light. Thus, it is conceivable that photobiology is integral to plant defense. Although several studies show an important role for light in plant defense [32], the precise molecular mechanisms underlying interactions between plant immunity and light perception are less understood. Here we show that COP1, an important master regulator that negatively regulates photomorphogenesis by degrading key proteins involved in light-regulated plant development, plays an equally important role in HRT-mediated defense against TCV and RPM1-mediated resistance against *avrRPM1* bacteria. However, in contrast to its role in photomorphogenesis, COP1 functions as a positive regulator in plant defense and is required for the stability of the R proteins HRT and RPM1. COP1 conferred regulation of HRT involves at least two DRB proteins, DRB1 and DRB4, which are well known components of the RNA silencing machinery. A mutation in COP1 results in the degradation of DRB1 and DRB4, which confer stability to HRT. Likewise, DRB4 is also required for the stability of RPM1 [10], and consistent with this result RPM1 accumulates to very low levels in the *cop1* plants.

Intriguingly, besides DRB1 and DRB4, three other DRB proteins also participate in TCV resistance by regulating levels of the R protein HRT. This corresponds to the physical interaction of HRT with DRB1, DRB3, DRB4, and DRB5. Normal levels of DRB2 in *cop1* plants suggests that COP1 might specifically regulate DRB1 and DRB4 proteins. We were unable to determine DRB3 and DRB5 levels in *cop1* plants due to lack of specific antibodies. The severely stunted phenotype of *cop1* in comparison to *drb1 drb4* double mutants suggests that additional components might contribute to the growth phenotype of *cop1* mutant plants (S6D Fig).

Normal photomorphogenic response displayed by the *drb* mutants suggests that the loss of DRB proteins does not alter COP1 function. Thus, COP1 likely protects DRB1 and DRB4 from one or more negative regulators that target these proteins for degradation (Fig 6D). Consequently, loss of COP1 would render these negative regulators active resulting in the degradation of DRB1 and DRB4. COP1 was recently shown to stabilize DRB1 by negatively regulating an unknown protease [24]. However, unlike DRB1, COP1 does not interact with DRB4 or APC10 E3 ligase, which negatively regulates DRB4. Thus, it is possible that COP1- and APC10-mediated regulation of DRB4 involves independent or indirect processes. Clearly, COP1 is epistatic to APC10 in relation to their effect on DRB4 since increased levels of APC10 was able to overcome COP1-mediated positive regulation of DRB4. Normal photomorphogenic response displayed by the APC10 overexpressing plants suggests that overexpression of APC10 does not alter COP1 function.

Interestingly, no physical interaction was observed between HRT and DRB2, even though the *drb2* mutant contains little or no HRT protein similar to the other *drb* mutants. This presents several possibilities: 1) DRB2 regulates HRT levels via its presence in HRT complexes comprising other DRB proteins. In fact, DRB2 and DRB4 were shown to interact with DRB1 and DRB5 in far-western assays [27] and supported by interactome analysis (S7A Fig). Although these interactions cannot be detected *in planta*, it is possible that they exist but are undetectable under the harsh conditions used for *in planta* IP assays. Weak interactions between DRB2 and DRB1/5 could enable the formation of multi-protein complexes that stabilize HRT. Loss of one or more components could disrupt such complexes resulting in the degradation of HRT. 2) DRB2 regulates HRT levels via its presence in HRT complexes comprising other proteins (other than DRBs; S7A Fig). Indeed, DRB2 is known to interact with other plant proteins including forming large molecular weight complexes and interacting with proteins involved in the regulation of chromatin functions [33]. Whether those proteins interact with HRT and/or affect its stability is not known. Similar to DRB2, DRB4 also forms a high molecular weight ~2 MDa complex [33] (S7B Fig). 3) DRB2 regulates HRT levels by negatively regulating a protein that degrades HRT. Notably, a high molecular weight complex comprising DRB2 contains MSI4, which functions as a substrate adaptor for CULLIN4 (CUL4)- Damaged DNA Binding Protein1 (DDB1) ubiquitin E3 ligases. Furthermore, CUL4-DDB1 interacts with the COP1 complex to regulate photomorphogenesis and flowering [34, 35], suggesting a potential link between DRB2 and the regulation of HRT levels via E3 ubiquitin ligases.

Contrary to previous studies that examined the subcellular localization of transiently expressed DRB1 in heterologous plants [26–28], we and Cho et al., [24] show that the bulk of transgene-expressed DRB1 is present in the cytosol of Arabidopsis plants. Notably, TCV infection relocalized ~2.34-fold DRB1 from the nucleus to cytosol. This is reminiscent of DRB4, which relocalizes from the nucleus to cytosol in the presence of TCV [10]. Interestingly, the extranuclear enrichment of DRB1 and DRB4 in TCV infected plants is associated with loss of interaction with HRT. Furthermore, even though both DRB1 and DRB4 can potentiate HRT-mediated cell death to TCV, only the *drb1* mutant is impaired in HR to TCV. Thus, DRB1 likely plays a key role in the activation of HRT while DRB4 has a minor role. Indeed, *HRT cop1* plants did not generate visible or microscopic HR against TCV. Thus, DRB1 and DRB4 act additively with DRB1 playing a major role in defense against TCV.

NBS-LRR proteins are multi-domain R proteins, which in the absence of pathogen infection remain in an inactive state. It is thought that the activated state of the R proteins involves conformational changes that exposes the N-terminal domain and thereby allows the R proteins to interact with their signaling partners [1]. For instance, activation of Rx was proposed to involve CP-mediated disruption of intramolecular interactions [36]. Similarly, R protein MLA in barley was shown to self-associate *in planta* in an effector-independent manner [37]. Our combined results, that HRT can self-associate [10], form complexes with DRB1 and DRB4 that are disrupted by CP, together with reduced stability of HRT in *drb1* and *drb4* backgrounds, propose a new model for R protein activation in plants. According to this model, DRB1 and DRB4 proteins help to maintain HRT in a dormant and stable state. CP-triggered dissociation of HRT-DRB complexes relieves the DRB1/4-mediated repression of HRT, facilitating a conformational change that triggers activation of HRT. It is possible that DRB2, 3 and 5 serve as decoys for CP and this further explains the inability of CP to disrupt the HRT-DRB3 interaction. Determining the precise relationships between the different DRB proteins in regulating various aspects of plant development will help better elucidate the canonical and non-canonical functions of these proteins.

## Materials and methods

### Plant growth conditions, genetic analysis and generation of transgenic plants

Plants were grown in MTPS 144 Conviron (Winnipeg, MB, Canada) walk-in-chambers at 22°C, 65% relative humidity and 14 hour photoperiod. The photon flux density of the day period was 106.9 μmoles m^-2^ s^-1^ and was measured using a digital light meter (Phytotronic Inc, Earth city, MO). Plants were grown on autoclaved Pro-Mix soil (Premier Horticulture Inc., PA, USA). Soil was fertilized once using Scotts Peter’s 20:10:20 peat lite special general fertilizer that contained 8.1% ammoniacal nitrogen and 11.9% nitrate nitrogen (Scottspro.com). Plants were irrigated using deionized or tap water.

Crosses were performed by emasculating the flowers of the recipient genotype and pollinating with the pollen from the donor. F2 plants showing the wt genotype at the mutant locus were used as controls in all experiments. The wt and mutant alleles were identified by PCR, CAPS, or dCAPS analysis. The Col-0-*HRT* line was generated after eight backcrosses of F1 derived from a Di-17 x Col-0 cross with Col-0, which was used as a recurrent parent. The F1 and F2 progenies from each backcross were genotyped for HRT and those from initial and final backcrosses were tested for HR and resistance phenotypes.

The Di-17 and Col-0 transgenic plants expressing HRT-Flag transgene are described earlier [15]. For transgenic overexpression of *DRBs*, the cDNA spanning the coding region were cloned into the pGWB5 vector [38], and expressed using 35S promoter and NOS terminator. The transgenic plants were selected on plates containing kanamycin (50 μg/ml) and hygromycin (17 μg/ml). For native expression of *DRBs*, the Myc or Flag-HA tagged DRBs along with their respective promoters were cloned into pCambia 1300 derived vector and transformed into respective *drb* mutant backgrounds. Genetic complementation was assayed by analyzing the levels of siRNA, as described before [33].

### RNA extraction, RNA gel-blot analyses and qRT-PCR

Small-scale extraction of RNA from two or three leaves (per sample) was performed with the TRIzol reagent (Invitrogen, CA), following the manufacturer’s instructions. RNA gel blot analysis and synthesis of random-primed probes for *PR-1*, *CP* and *DRB4* were carried out as described previously [14].

RNA quality and concentration were determined by gel electrophoresis and determination of A_260_. Reverse transcription (RT) and first strand cDNA synthesis were carried out using Superscript II (Invitrogen, CA). Quantitative RT-PCR was carried out as described before [39]. Each sample was run in triplicates and *ACTIN II* (At3g18780) expression levels were used as internal control for normalization. Cycle threshold values were calculated by SDS 2.3 software.

### Trypan-blue staining

The leaves were vacuum-infiltrated with trypan-blue stain prepared in 10 mL acidic phenol, 10 mL glycerol, and 20 mL sterile water with 10 mg of trypan blue. The samples were placed in a heated water bath (90°C) for 2 min and incubated at room temperature for 2–12 h. The samples were destained using chloral hydrate (25 g/10 mL sterile water; Sigma), mounted on slides and observed for cell death with a compound microscope. The samples were photographed using an AxioCam camera (Zeiss, Germany) and images were analyzed using Openlab 3.5.2 (Improvision) software.

### Pathogen infections

Transcripts synthesized *in vitro* from a cloned cDNA of TCV using T7 RNA polymerase were used for viral infections. For inoculations, the viral transcript was suspended at a concentration of 0.05 μg/ μL in inoculation buffer, and the inoculation was performed as described earlier [40]. After viral inoculations, the plants were transferred to a Conviron MTR30 reach-in chamber maintained at 22°C, 65% relative humidity and 14 hour photoperiod. HR was determined visually three-to-four days post-inoculation (dpi). Resistance and susceptibility was scored at 14 to 21 dpi and confirmed by northern- or western-gel blot analysis. Susceptible plants showed stunted growth, crinkling of leaves and drooping of the bolt.

The bacterial strain pVSP61 (empty vector), or *avrRpm1* were grown overnight in King’s B medium containing rifampicin and kanamycin (Sigma, MO). The bacterial cells were harvested, washed and suspended in 10 mM MgCl_2_. The cells were diluted to a final density of 10^5^ or 10^6^ CFU/mL (A_600_) and used for infiltration. The bacterial suspension was injected into the abaxial surface of the leaf using a needle-less syringe. Three leaf discs from the inoculated leaves were collected at 0 and 3 or 6 dpi. The leaf discs were homogenized in 10 mM MgCl_2_, diluted 10^3^ or 10^4^ fold and plated on King’s B medium.

### Protein extraction, immunoblot analysis and nuclear fractionation

Proteins were extracted in buffer containing 50 mM Tris-HCl, pH7.5, 10% glycerol, 150 mM NaCl, 10 mM MgCl_2_, 5 mM EDTA, 5 mM DTT, and 1 X protease inhibitor cocktail (Sigma-Aldrich, St. Louis, MO). Protein concentration was measured by the Bio-RAD protein assay (Bio-Rad, CA). For small scale extractions 2–3 leaves were homogenized per sample.

For Ponceau-S staining, PVDF membranes were incubated in Ponceau-S solution (40% methanol (v/v), 15% acetic acid (v/v), 0.25% Ponceau-S). The membranes were destained using deionized water.

Proteins (30–50 μg) were fractionated on a 7–10% SDS-PAGE gel and subjected to immunoblot analysis using α-CP, α-Myc, α-Flag (Sigma-Aldrich, St. Louis, MO) or α-GFP antibody. Immunoblots were developed using ECL detection kit (Roche) or alkaline-phosphatase-based color detection. Fold change, normalized with Rubisco, Actin or H3 proteins, in western blots was quantified using Image Quant software.

Coimmunoprecipitations were carried out as described earlier [13, 15]. Briefly, ~1 g of infiltrated leaf tissues were harvested and extracted in buffer containing 10% (v/v) glycerol, 25 mM Tris-HCL pH 7.5, 1 mM EDTA, 150 mM NaCl, 2% (w/v) polyvinylpolypyrrolidone and 1 X protease inhibitor cocktail. Extracts were centrifuged twice at 12,000 g for 10 min at 4°C and supernatant was incubated overnight with 20 μl of anti-Flag M2 or anti-Myc affinity beads (Sigma-Aldrich, St. Louis, MO). Beads were washed three times with the extraction buffer and the proteins were fractionated on SDS-PAGE gels as described above.

Nuclear fractionation was carried out as described before [41].

### Size exclusion chromatography

For gel filtration experiments, ground mixed flower tissues were dissolved in lysis buffer (150 mM NaCl, 0,1% Igepal, 50 mM Tris pH8, 5 mM MgCl2, 10 μM MG132 1X protease inhibitor cocktail). Supernatant was filtered through 0.45 μm membrane, and further processed by a 2 hours centrifugation, 4500 rpm on Amicon Ultra centrifugal units (Millipore). 500μl of the resulting crude extract was loaded onto the Superose 6 10/200 column (GE Healthcare) to perform size exclusion chromatography, 500μl/minute, and 500μl fractions were collected, precipitated separately in 2 volumes of absolute Ethanol overnight at 4°C, and pellets were resuspended in 100ul 2X Laemmli buffer. Separation and blotting was then performed as described above. Size markers were run in similar settings, in a separate run.

### Confocal microscopy

For confocal imaging, samples were scanned on an Olympus FV1000 microscope (Olympus America, Melvile, NY). GFP (YFP), and RFP were excited using 488, and 543 nm laser lines, respectively. Constructs were made using pSITE [42], pEarlyGate or pGWB based binary vectors using Gateway technology and introduced in *A*. *tumefaciens* strain LBA4404 for agroinfiltration into *N*. *benthamiana* and MP90 for Arabidopsis transformation. Agrobacterium strains carrying various constructs were infiltrated into wild-type or transgenic *N*. *benthamiana* plants expressing CFP-tagged nuclear protein H2B. 48 h later, water-mounted sections of leaf tissue were examined by confocal microscopy using a water immersion PLAPO60XWLSM 2 (NA 1.0) objective on a FV1000 point-scanning/point-detection laser scanning confocal 3 microscope (Olympus) equipped with lasers spanning the spectral range of 405–633 nm. RFP, CFP and GFP/YFP overlay images (40X magnification) were acquired at a scan rate of 10 ms/pixel. Images were acquired sequentially when multiple fluorophores were used. Olympus FLUOVIEW 1.5 was used to control the microscope, image acquisition and the export of TIFF files.

## Supporting information

S1 Fig (A) Quantitative RT-PCR analysis showing relative levels of *RPM1* transcript in wild-type (Col-0) and *cop1* mutant plants. This experiment was repeated twice using two or more independent cDNA preparations as templates. (**B**) Confocal micrographs showing BiFC for RPM1 and COP1. Agroinfiltration was used to express protein in transgenic *N*. *benthamiana* plants expressing the nuclear marker CFP-H2B (Scale bar, 10 μM). Arrows indicate nucleus. All interactions were confirmed using both combinations of reciprocal N-EYFP/C-EYFP fusion proteins in three separate experiments (three replicates per experiment). (**C**) Western blots showing relative levels of DRB2 in flowers from indicated genotypes. Ponceau-S staining of the Western blots was used as the loading control. Arrows indicate the target protein corresponding to the indicated antibody. This experiment was repeated three times with similar results.
(TIF)

S2 Fig DRB proteins are required for HRT-mediated resistance. (**A**) Typical morphological phenotypes of four-week-old soil grown *drb* mutants plants. (**B**) Typical morphological phenotypes of TCV inoculated Di-17, *HRT drb* and Col-0 plants. Plants were photographed at 18 dpi.
(TIF)

S3 Fig HRT interacts with DRB1, DRB3 and DRB5 but not DRB2. (**A**) Western blot showing relative HRT levels in F2 plants derived from Col-0-HRT x *drb* cross segregating for DRB1 (left panel) or DRB2 (right panel). Ponceau-S staining of the western blot was used as the loading control. This experiment was repeated with multiple F2 plants with similar results. (**B**) Quantitative RT-PCR analysis showing relative levels of *HRT-FLAG* transcript in *drb* mutant background. This experiment was repeated twice using two or more independent cDNA preparations as templates. (**C**) Typical morphological phenotypes of *hrt drb* and Col-0-*HRT* plants inoculated with TCV. Leaves were photographed at 10 dpi. (**D**) Confocal micrographs showing bi-molecular fluorescence complementation (BiFC) for indicated proteins. Agroinfiltration was used to express protein in transgenic *N*. *benthamiana* plants expressing the nuclear marker CFP-H2B (Scale bar, 10 μM). Arrow indicates nucleus. All interactions were confirmed using both combinations of reciprocal N-EYFP/C-EYFP fusion proteins in three separate experiments (three replicates per experiment). (**E-H**) Co-immunoprecipitation (IP) of DRB1-Myc (**E**), DRB2-Myc (**F**), DRB3-Myc (**G**) and DRB5-Myc (**H**) with HRT-Flag. *N*. *benthamiana* plants were agroinfiltrated and immunoprecipitated proteins were analyzed with α-Myc and α-Flag. HRT and DRB proteins were expressed under 35S promoter. This experiment was repeated twice with similar results. (**I**) Electrolyte leakage in *N*. *benthamiana* leaves infiltrated with buffer (150 μM acetosyringone, 10 mM MES, 10 mM MgCl_2_, pH 5.6), or Agrobacterium cultures (suspended in the same buffer) expressing HRT, HRT+CP, HRT+CP+DRB or HRT+CP+EDS1. Error bars represent SD (n = 6). (**J**) Quantitative RT-PCR analysis showing relative levels of *DRB* transcripts in transgenic Di-17 plants overexpressing *DRB1*, *DRB3* or *DRB4*. This experiment was repeated twice using two independent cDNA preparations as templates. Error bars indicate SD. Asterisks indicate data statistically significant from that of control (Di-17) (P<0.05, n = 4). (**K**) HR formation in TCV-inoculated Di-17 and transgenic plants showing reduced- (line#1) or over-expression (line#6) of *DRB1*. The HR phenotype was evaluated in ~20 plants that were analyzed in two separate experiments. (**L**) Typical morphological phenotypes of TCV inoculated Di-17, Col-0 and transgenic plant showing reduced- (#1) or over-expression (#6) of *DRB1*. Plants were photographed at 18 dpi. (**M**) Percentage of resistant plants observed among indicated genotypes. The susceptible plants were scored based on crinkled and short bolt phenotypes. (**N**) Western blot showing relative levels of CP in the systemic bolt tissues of Di-17, Col-0 and three independent *35S-DRB1-1* plants. The plants were sampled at 14 dpi. Ponceau-S staining of the western blot was used as the loading control. This experiment was repeated three times with similar results. (**O & P**) Typical morphological phenotypes of Di-17 and *35S-DRB* plants inoculated with a virulent R8A strain of TCV. Leaves (**O**) and plants (**P**) were photographed at 3 and 18 dpi, respectively. (**Q**) Western blot showing relative levels of TCV CP in Di-17 and *35S-DRB* plants generated in Di-17 background. The inoculated leaves were sampled at 3 dpi. Ponceau-S staining of the western blot was used as the loading control. This experiment was repeated two times with similar results.
(TIF)

S4 Fig The *drb* mutants show wild-type-like photomorphogenesis. (**A**) Typical growth phenotypes seen in wild-type (Col-0), *cop1*, and *drb* mutants grown under light (upper panel) or dark (lower panel) conditions. (**B**) Hypocotyl length in Col-0, *cop1*, and *drb* mutants grown under dark condition. These experiments were repeated twice with similar results.
(TIF)

S5 Fig DRB proteins interact with CP and DRB1 relocalizes to cytosol when coexpressed with CP. (**A and B**) Confocal micrographs showing localization of CP-RFP and DRB-GFP expressed individually (**A**) or coexpressed (**B**) in *N*. *benthamiana*. Arrows and arrowheads indicate nucleus and inclusion structures, respectively (Scale bars, 10 μM). This experiment was repeated three times (three replicates per experiment) with similar results. (**C-F**) Co-IP of DRB1-Flag (**C**), DRB2-Flag (**D**) and DRB3-Flag (**E**) and DRB5-Flag (**F**) with CP. *N*. *benthamiana* plants were agroinfiltrated and immunoprecipitated proteins were analyzed with α-CP and α-Flag. HRT and DRB proteins were expressed under 35S promoter. This experiment was repeated twice with similar results. (**G-H**) Yeast-two hybrid assay showing interaction between CP and DBR4. **G** shows growth on selection medium and **H** shows β-glactosidase assay. Yeast colonies co-expressing bait (pGADT7) and prey (pGBKT7) plasmids were streaked on plates without (-) leucine and tryptophan or without leucine, tryptophan, and histidine. (**I**) Confocal micrographs showing BiFC for indicated proteins. Agroinfiltration was used to express protein in transgenic *N*. *benthamiana* plants expressing the nuclear marker CFP-H2B (Scale bar, 10 μM). Arrows and arrowheads indicate nucleus and inclusion structures, respectively. All interactions were confirmed using both combinations of reciprocal N-EYFP/C-EYFP fusion proteins in three separate experiments (three replicates per experiment). (**J**) Confocal micrographs showing localization of indicated proteins. Agroinfiltration was used to express protein in transgenic *N*. *benthamiana* plants expressing the nuclear marker CFP-H2B (Scale bar, 10 μM). Arrows indicate nucleus. (**K**) Co-IP of DRB3-Myc or DRB4-Myc with HRT-Flag in the presence or absence of CP. *N*. *benthamiana* plants were agroinfiltrated and immunoprecipitated proteins were analyzed with α-Myc and α-Flag. This experiment was repeated two times with similar results.
(TIF)

S6 Fig The *APC* overexpressing plants show normal photomorphogenesis. (**A**) Confocal micrographs showing BiFC for indicated proteins. Agroinfiltration was used to express protein in transgenic *N*. *benthamiana* plants expressing the nuclear marker CFP-H2B (Scale bar, 10 μM). Arrows indicate nucleus. All interactions were confirmed using both combinations of reciprocal N-EYFP/C-EYFP fusion proteins in three separate experiments (three replicates per experiment). (**B**) Hypocotyl length in Col-0, *cop1*, and *APC10* overexpressing (OE) plants grown under dark condition. These experiments were repeated twice with similar results. (**C**) Model showing proteins interacting with COP1, APC10 and DRB4. This model includes both predicted and confirmed interactions and was created using Arabidopsis Interaction viewer. (**D**) Typical morphological phenotypes of soil grown four-week-old Col-0, *cop1* and *drb1 drb4* plants.
(TIF)

S7 Fig DRB2 and DRB4 form high molecular weight complexes. (**A**) Model showing relationship between DRB interactomes. The model includes both predicted and confirmed interactions and was created using Arabidopsis Interaction viewer. The program did not predict any interactions for DRB3. (**B**) Distribution profile of DRB4-Myc and DRB2-FlagHA proteins, after size exclusion chromatography on a Superose 6 column. Both are detected in high molecular weight complexes, superior to 669 kDa. Five hundred microliter fractions were collected, precipitated and equivalent amounts were analyzed by western blot. Four separate gels were used to analyze one profile. Signals were detected with Myc and HA antibodies.
(TIF)

S1 Table Mutant backgrounds used to express tagged *DRB* genes under their respective promoters. (DOCX)

S2 Table Epistatic analysis of F2 population derived from crosses between Di-17 and various wild-type or mutant lines. (DOCX)
