# Supplementary figures and images for: COP1, a negative regulator of photomorphogenesis, positively regulates plant disease resistance via double-stranded RNA binding proteins
Source: PLoS Pathog. 2018 Mar 7;14(3):e1006894. doi: 10.1371/journal.ppat.1006894 (PMC5871017; doi:10.1371/journal.ppat.1006894)

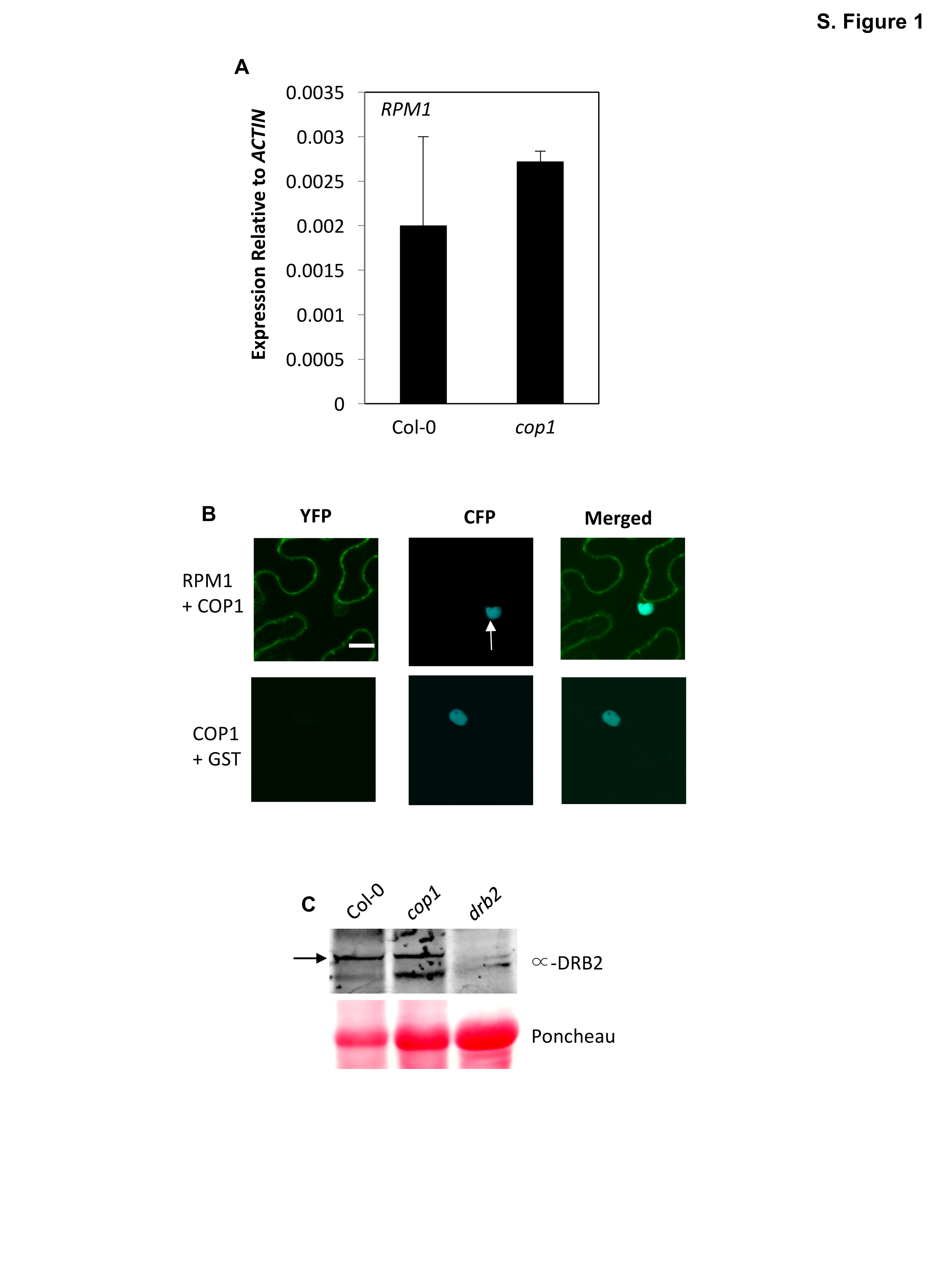

Supplement: S1 Fig — (A) Quantitative RT-PCR analysis showing relative levels of RPM1 transcript in wild-type (Col-0) and cop1 mutant plants. This experiment was repeated twice using two or more independent cDNA preparations as templates. (B) Confocal micrographs showing BiFC for RPM1 and COP1. Agroinfiltration was used to express protein in transgenic N. benthamiana plants expressing the nuclear marker CFP-H2B (Scale bar, 10 μM). Arrows indicate nucleus. All interactions were confirmed using both combinations of reciprocal N-EYFP/C-EYFP fusion proteins in three separate experiments (three replicates per experiment). (C) Western blots showing relative levels of DRB2 in flowers from indicated genotypes. Ponceau-S staining of the Western blots was used as the loading control. Arrows indicate the target protein corresponding to the indicated antibody. This experiment was repeated three times with similar results. (TIF) [file ppat.1006894.s001.tif]

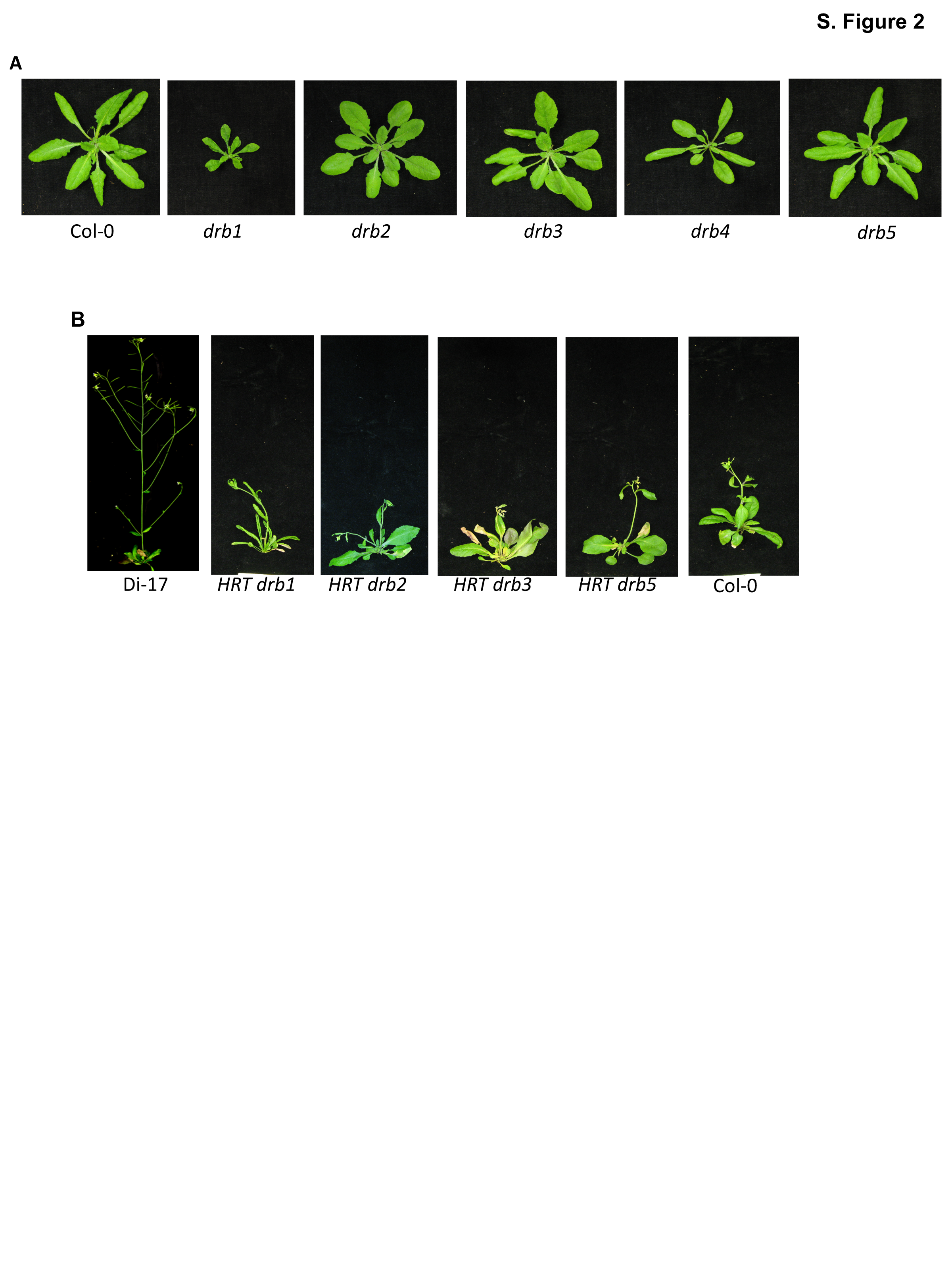

Supplement: S2 Fig — (A) Typical morphological phenotypes of four-week-old soil grown drb mutants plants. (B) Typical morphological phenotypes of TCV inoculated Di-17, HRT drb and Col-0 plants. Plants were photographed at 18 dpi. (TIF) [file ppat.1006894.s002.tif]

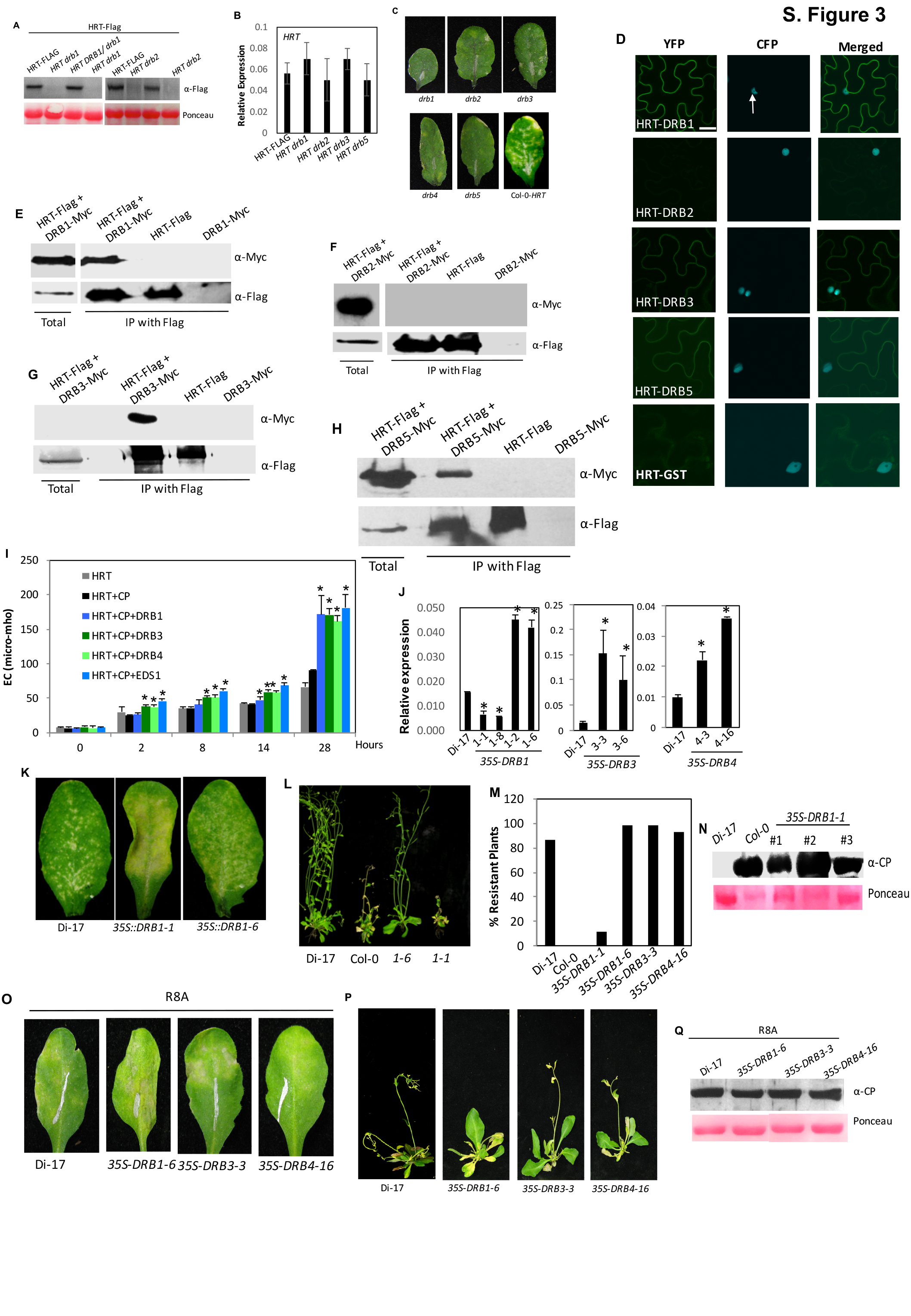

Supplement: S3 Fig — (A) Western blot showing relative HRT levels in F2 plants derived from Col-0-HRT x drb cross segregating for DRB1 (left panel) or DRB2 (right panel). Ponceau-S staining of the western blot was used as the loading control. This experiment was repeated with multiple F2 plants with similar results. (B) Quantitative RT-PCR analysis showing relative levels of HRT-FLAG transcript in drb mutant background. This experiment was repeated twice using two or more independent cDNA preparations as templates. (C) Typical morphological phenotypes of hrt drb and Col-0-HRT plants inoculated with TCV. Leaves were photographed at 10 dpi. (D) Confocal micrographs showing bi-molecular fluorescence complementation (BiFC) for indicated proteins. Agroinfiltration was used to express protein in transgenic N. benthamiana plants expressing the nuclear marker CFP-H2B (Scale bar, 10 μM). Arrow indicates nucleus. All interactions were confirmed using both combinations of reciprocal N-EYFP/C-EYFP fusion proteins in three separate experiments (three replicates per experiment). (E-H) Co-immunoprecipitation (IP) of DRB1-Myc (E), DRB2-Myc (F), DRB3-Myc (G) and DRB5-Myc (H) with HRT-Flag. N. benthamiana plants were agroinfiltrated and immunoprecipitated proteins were analyzed with α-Myc and α-Flag. HRT and DRB proteins were expressed under 35S promoter. This experiment was repeated twice with similar results. (I) Electrolyte leakage in N. benthamiana leaves infiltrated with buffer (150 μM acetosyringone, 10 mM MES, 10 mM MgCl2, pH 5.6), or Agrobacterium cultures (suspended in the same buffer) expressing HRT, HRT+CP, HRT+CP+DRB or HRT+CP+EDS1. Error bars represent SD (n = 6). (J) Quantitative RT-PCR analysis showing relative levels of DRB transcripts in transgenic Di-17 plants overexpressing DRB1, DRB3 or DRB4. This experiment was repeated twice using two independent cDNA preparations as templates. Error bars indicate SD. Asterisks indicate data statistically significant from that of control (Di-17) (P< [file ppat.1006894.s003.tif]

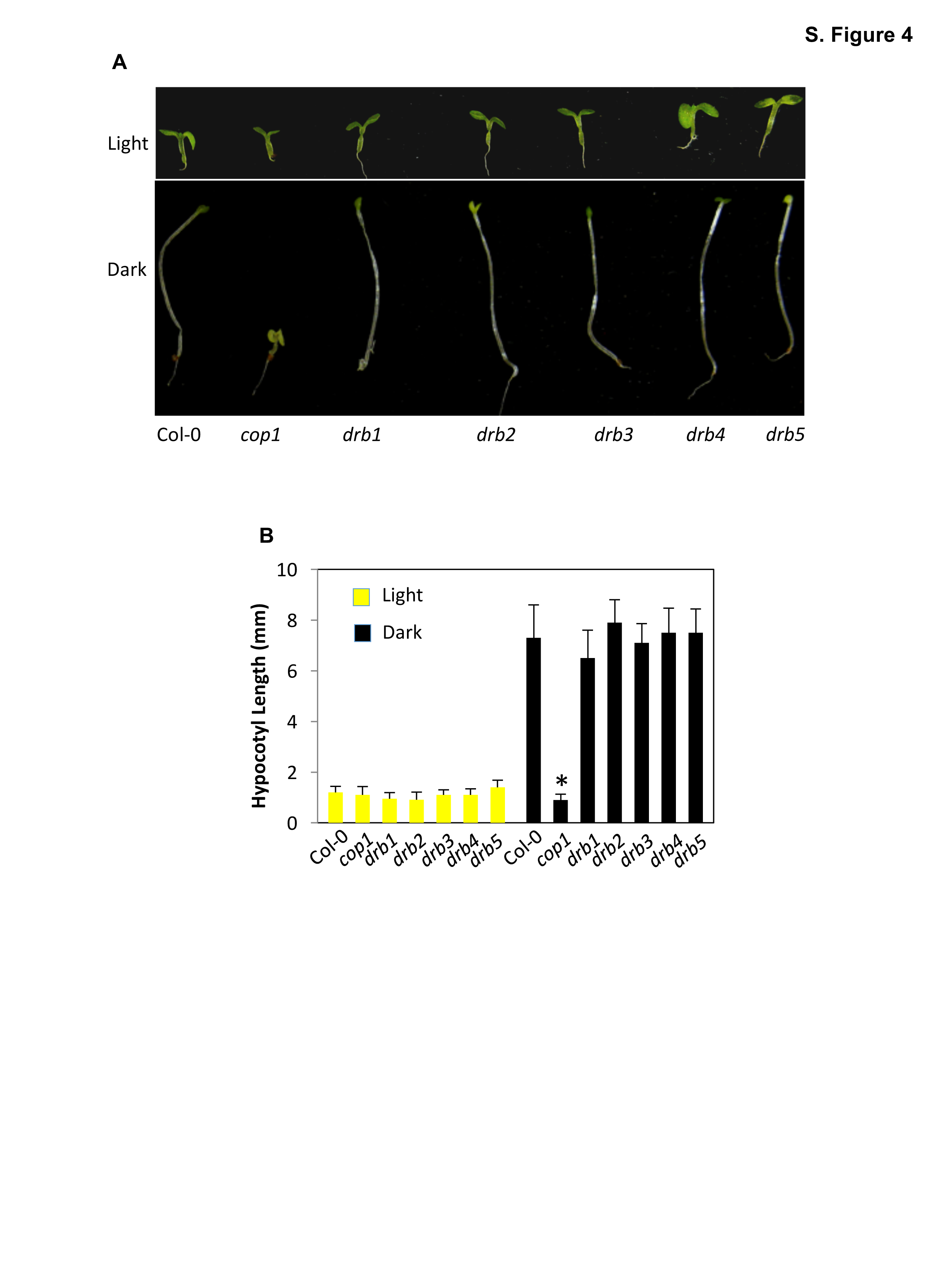

Supplement: S4 Fig — (A) Typical growth phenotypes seen in wild-type (Col-0), cop1, and drb mutants grown under light (upper panel) or dark (lower panel) conditions. (B) Hypocotyl length in Col-0, cop1, and drb mutants grown under dark condition. These experiments were repeated twice with similar results. (TIF) [file ppat.1006894.s004.tif]

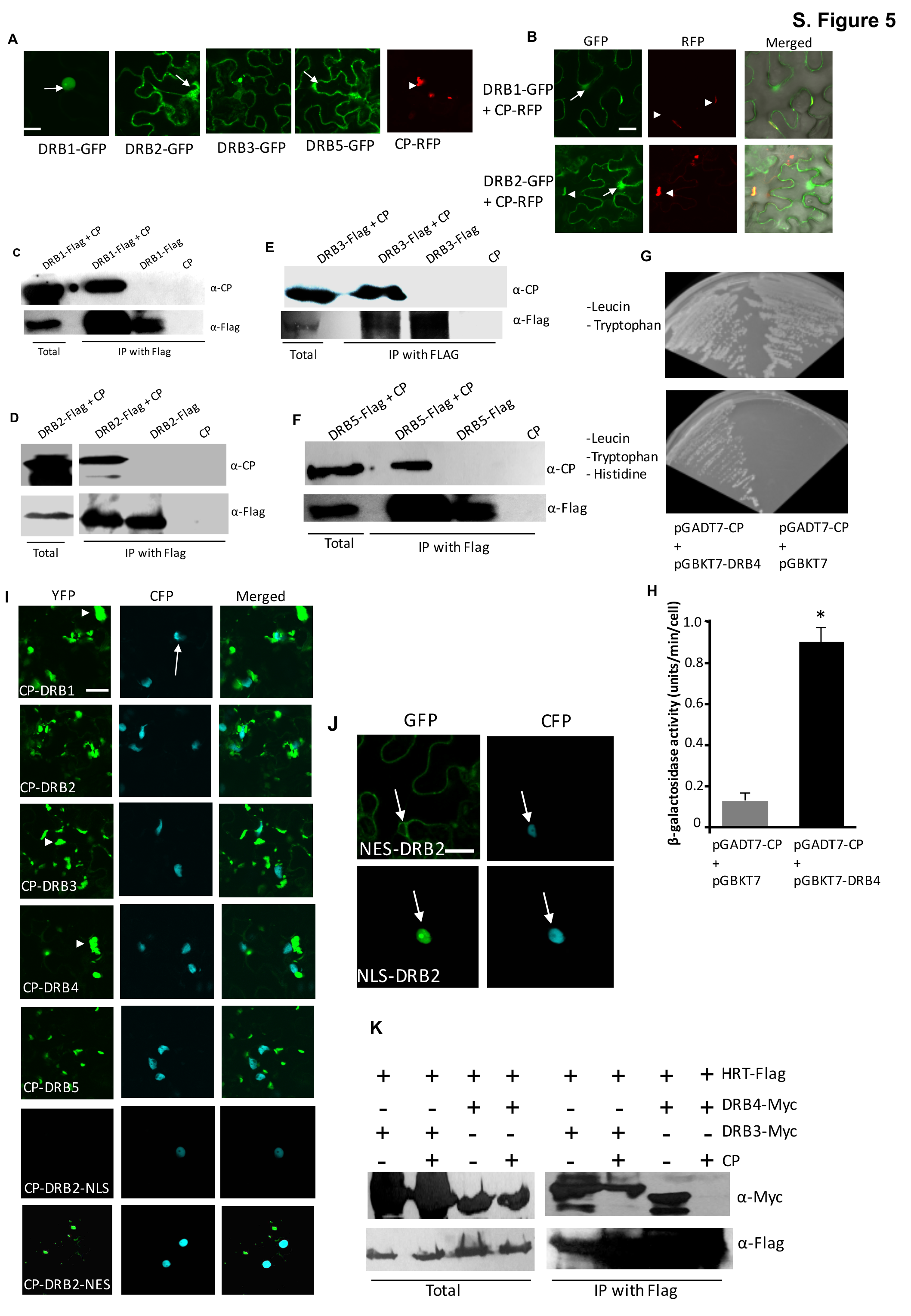

Supplement: S5 Fig — (A and B) Confocal micrographs showing localization of CP-RFP and DRB-GFP expressed individually (A) or coexpressed (B) in N. benthamiana. Arrows and arrowheads indicate nucleus and inclusion structures, respectively (Scale bars, 10 μM). This experiment was repeated three times (three replicates per experiment) with similar results. (C-F) Co-IP of DRB1-Flag (C), DRB2-Flag (D) and DRB3-Flag (E) and DRB5-Flag (F) with CP. N. benthamiana plants were agroinfiltrated and immunoprecipitated proteins were analyzed with α-CP and α-Flag. HRT and DRB proteins were expressed under 35S promoter. This experiment was repeated twice with similar results. (G-H) Yeast-two hybrid assay showing interaction between CP and DBR4. G shows growth on selection medium and H shows β-glactosidase assay. Yeast colonies co-expressing bait (pGADT7) and prey (pGBKT7) plasmids were streaked on plates without (-) leucine and tryptophan or without leucine, tryptophan, and histidine. (I) Confocal micrographs showing BiFC for indicated proteins. Agroinfiltration was used to express protein in transgenic N. benthamiana plants expressing the nuclear marker CFP-H2B (Scale bar, 10 μM). Arrows and arrowheads indicate nucleus and inclusion structures, respectively. All interactions were confirmed using both combinations of reciprocal N-EYFP/C-EYFP fusion proteins in three separate experiments (three replicates per experiment). (J) Confocal micrographs showing localization of indicated proteins. Agroinfiltration was used to express protein in transgenic N. benthamiana plants expressing the nuclear marker CFP-H2B (Scale bar, 10 μM). Arrows indicate nucleus. (K) Co-IP of DRB3-Myc or DRB4-Myc with HRT-Flag in the presence or absence of CP. N. benthamiana plants were agroinfiltrated and immunoprecipitated proteins were analyzed with α-Myc and α-Flag. This experiment was repeated two times with similar results. (TIF) [file ppat.1006894.s005.tif]

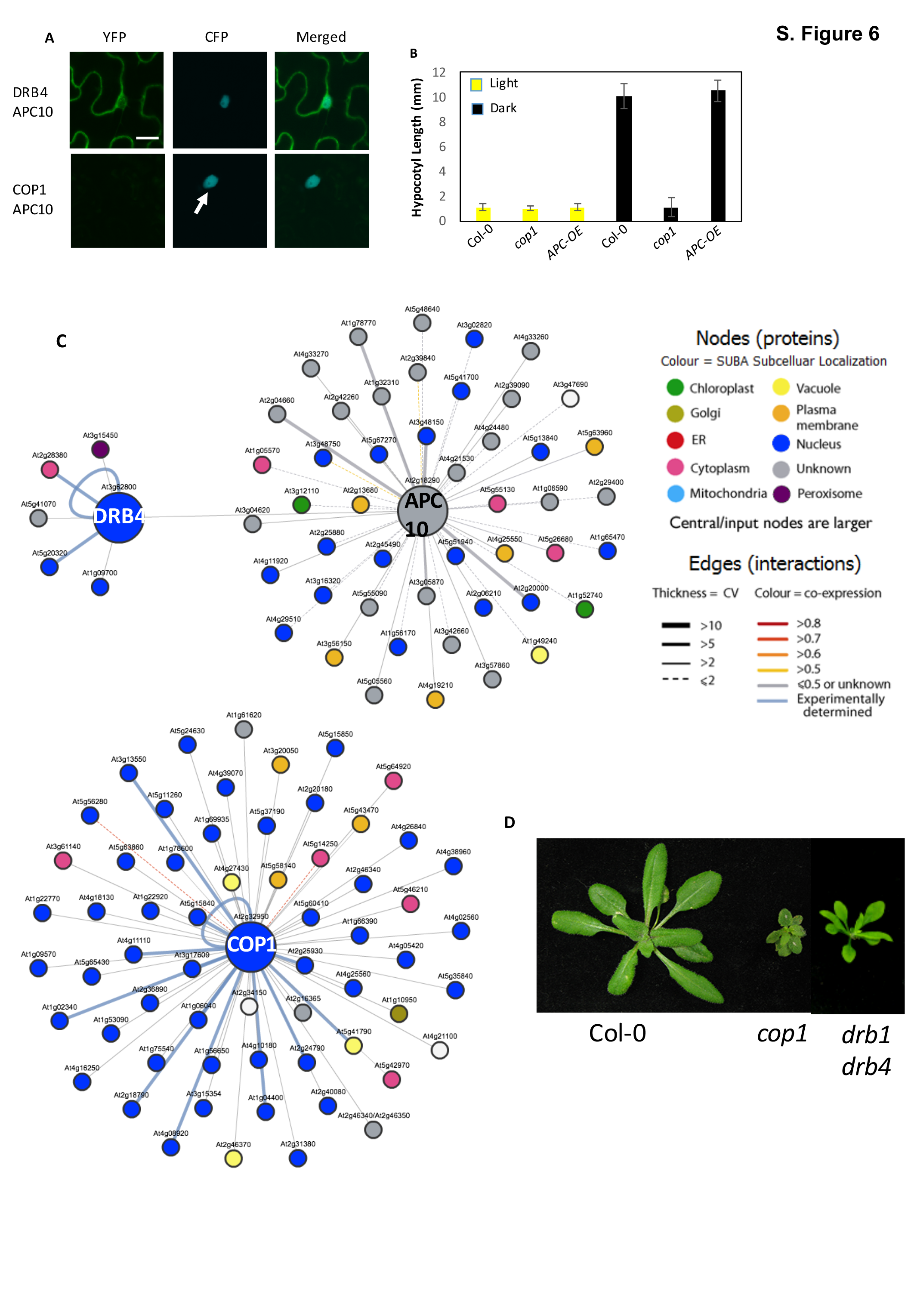

Supplement: S6 Fig — (A) Confocal micrographs showing BiFC for indicated proteins. Agroinfiltration was used to express protein in transgenic N. benthamiana plants expressing the nuclear marker CFP-H2B (Scale bar, 10 μM). Arrows indicate nucleus. All interactions were confirmed using both combinations of reciprocal N-EYFP/C-EYFP fusion proteins in three separate experiments (three replicates per experiment). (B) Hypocotyl length in Col-0, cop1, and APC10 overexpressing (OE) plants grown under dark condition. These experiments were repeated twice with similar results. (C) Model showing proteins interacting with COP1, APC10 and DRB4. This model includes both predicted and confirmed interactions and was created using Arabidopsis Interaction viewer. (D) Typical morphological phenotypes of soil grown four-week-old Col-0, cop1 and drb1 drb4 plants. (TIF) [file ppat.1006894.s006.tif]

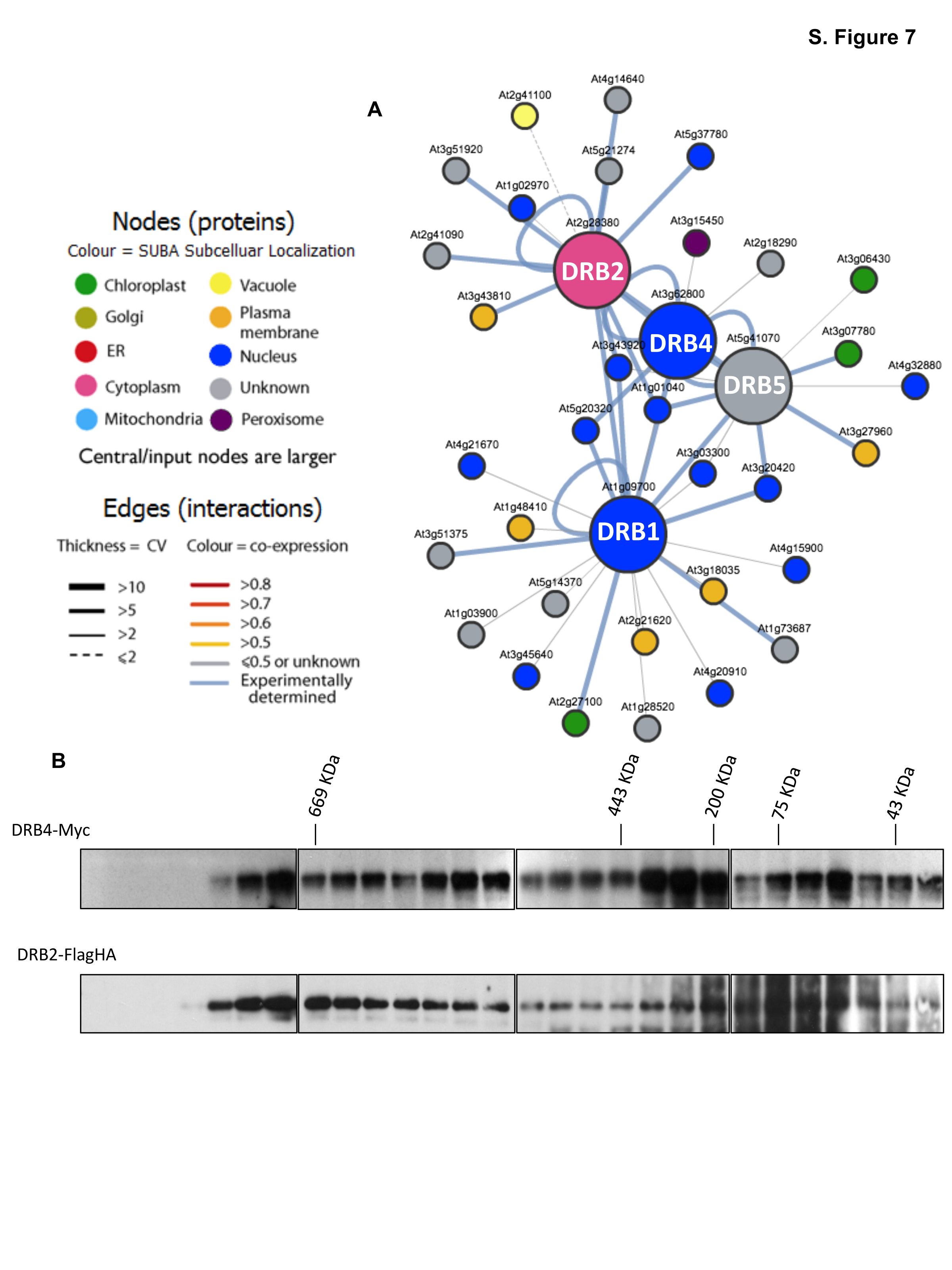

Supplement: S7 Fig — (A) Model showing relationship between DRB interactomes. The model includes both predicted and confirmed interactions and was created using Arabidopsis Interaction viewer. The program did not predict any interactions for DRB3. (B) Distribution profile of DRB4-Myc and DRB2-FlagHA proteins, after size exclusion chromatography on a Superose 6 column. Both are detected in high molecular weight complexes, superior to 669 kDa. Five hundred microliter fractions were collected, precipitated and equivalent amounts were analyzed by western blot. Four separate gels were used to analyze one profile. Signals were detected with Myc and HA antibodies. (TIF) [file ppat.1006894.s007.tif]
