# Supplementary material for: COP1, a negative regulator of photomorphogenesis, positively regulates plant disease resistance via double-stranded RNA binding proteins
Source: PLoS Pathog. 2018 Mar 7;14(3):e1006894. doi: 10.1371/journal.ppat.1006894 (PMC5871017; doi:10.1371/journal.ppat.1006894)
Supplement: S1 Table — (DOCX) [file ppat.1006894.s008.docx]

**Table S1.** Mutant backgrounds used to express tagged *DRB* genes under their respective promoters.

| **Gene** | **Allele name** | **T-DNA insertion line** | **Insertion site** | **ORF** |
| --- | --- | --- | --- | --- |
| ***DRB1*** | *drb1-2* | Salk_064863 | 699 | 236-2394 |
| ***DRB2*** | *drb2-1* | GABI_348A09 | 377 | 266-2083 |
| ***DRB3*** | *drb3-1* | Salk_00331 | 1189 | 361-1764 |
| ***DRB4*** | *drb4-1* | Salk_000736 | 313 | 350-1764 |
| ***DRB5*** | *drb5-1* | Salk_031307 | 1406 | 241-1610 |
