# Supplementary material for: COP1, a negative regulator of photomorphogenesis, positively regulates plant disease resistance via double-stranded RNA binding proteins
Source: PLoS Pathog. 2018 Mar 7;14(3):e1006894. doi: 10.1371/journal.ppat.1006894 (PMC5871017; doi:10.1371/journal.ppat.1006894)
Supplement: S2 Table — (DOCX) [file ppat.1006894.s009.docx]

**Table S2.** Epistatic analysis of F2 population derived from crosses between Di-17 and various wild-type or mutant lines.

| **Cross** | **Genotype ^a^** | **Plants obtained** | **HR ^b^** | **R ^c^** | **S ^d^** | **χ2** ^e^ | P |
| --- | --- | --- | --- | --- | --- | --- | --- |
| Di-17 x Col-0 | *HRT/-* | 129 | + | 29 | 100 | 0.43 | 0.50 |
| Di-17 x  *cop1* | *HRT/- cop1**HRT/- COP1/-* | 35  112 | -  + | 0  32 | 35  80 | 11.6  0.76 | 0.0006 ^f^  0.38 |
| Di-17 x  *drb1* | *HRT/- drb1**HRT/- DRB1/-* | 75  255 | -  + | 7  70 | 68  185 | 9.8  1.26 | 0.001 ^f^  0.26 |
| Di-17 x  *drb2* | *HRT/- drb2**HRT/- DRB2/-* | 68  247 | +  + | 2  55 | 66  192 | 17.64  0.98 | 0.001 ^f^  0.32 |
| Di-17 x  *drb3* | *HRT/- drb3**HRT/- DRB3/-* | 68  201 | +  + | 0  46 | 68  155 | 17.6  0.47 | 0.0001 ^f^  0.48 |
| Di-17 x  *drb5* | *HRT/- drb5* *HRT/- DRB5/-* | 8  177 | +  + | 0  21 | 8  73 | 2.66  1.37 | 0.10  0.24 |

^a^ The genotype at *HRT* and various mutant loci was determined by CAPS analysis

^b^ HR, hypersensitive response

^c^ Resistant

^d^ Susceptible

^e^ One degree of freedom; based on segregation of 3 susceptible:1 resistant plants

^f^ Statistically significant
